# Exploring novel secondary metabolites from natural products using pre-processed mass spectral data

**DOI:** 10.1038/s41598-019-54078-1

**Published:** 2019-11-22

**Authors:** Hyun Woo Kim, Seong Yeon Choi, Hyeon Seok Jang, Byeol Ryu, Sang Hyun Sung, Heejung Yang

**Affiliations:** 1 College of Pharmacy and Research Institute of Pharmaceutical Sciences, Seoul National University, Seoul, 08826 Korea; 2 Laboratory of Natural Products Chemistry, College of Pharmacy, Kangwon National University, Chuncheon, 24341 Korea

**Keywords:** Chromatography, Mass spectrometry, Secondary metabolism

## Abstract

Many natural product chemists are working to identify a wide variety of novel secondary metabolites from natural materials and are eager to avoid repeatedly discovering known compounds. Here, we developed liquid chromatography/mass spectrometry (LC/MS) data-processing protocols for assessing high-throughput spectral data from natural sources and scoring the novelty of unknown metabolites from natural products. This approach automatically produces representative MS spectra (RMSs) corresponding to single secondary metabolites in natural sources. In this study, we used the RMSs of *Agrimonia pilosa* roots and aerial parts as models to reveal the structural similarities of their secondary metabolites and identify novel compounds, as well as isolation of three types of nine new compounds including three pilosanidin- and four pilosanol-type molecules and two 3-hydroxy-3-methylglutaryl (HMG)-conjugated chromones. Furthermore, we devised a new scoring system, the Fresh Compound Index (FCI), which grades the novelty of single secondary metabolites from a natural material using an in-house database constructed from 466 representative medicinal plants from East Asian countries. We expect that the FCIs of RMSs in a sample will help natural product chemists to discover other compounds of interest with similar chemical scaffolds or novel compounds and will provide insights relevant to the structural diversity and novelty of secondary metabolites in natural products.

## Introduction

Natural products have been used for coping with illness and treating diseases for a long time; these compounds have been intensively studied and serve as a source of molecular diversity and inspiration for natural product chemists. Natural products are frequently identified as a rich source of chemical diversity for pharmaceutical lead or novel compound discovery; however, the rediscovery of known structures is a serious challenge for natural product researchers. In addition, even though the number of annually reported new compounds has been increasing, the chemical diversity of those compounds has been decreasing^1^. In recent years, mass spectrometry (MS) has been recognized as a state-of-the-art analytical technique that can provide substantial amounts of information for the high-throughput discovery of lead compounds from natural materials^2,3^. High-resolution MS (HRMS) analysers such as quadrupole time-of-flight (qTOF) and orbitrap are able to provide higher *m/z* resolution, larger dynamic range and better sensitivity, which are features that facilitate analysis of the structural properties of metabolites from natural sources^4–6^. Although it is easy to obtain spectral data from high-throughput MS experiments, the numerous ion peaks from raw MS spectral data should be pre-processed to acquire the *m/z* and intensity values of the parent and fragment ion peaks for interpreting the molecular structure, which are necessary to interpret the most likely structure from raw MS spectral data; exceptions include unwanted values, such as noise signals^7–9^, overlapped peaks and adduct ions^10–17^. After data pre-processing, the resulting MS spectral information has been used to identify known metabolites and to predict the structures of unknown compounds in natural products chemistry^18–22^. To identify and assign experimental MS spectra, many MS spectral databases filled with data from tens of thousands of small molecules are freely available online^22–25^. Despite the introduction of WEIZMASS, an MS spectral database built on more than 3,300 authentic standards in plants^26^, many public databases are still limited to primary metabolites from human samples or simple secondary metabolites from a few natural materials, and the databases do not cover the vast number of secondary metabolites in natural products. Recently, a few *in silico* fragmentation databases, such as ISDB^27^ and CSI:FingerID^18^, were introduced for use in dereplication studies and in the identification of secondary metabolites in natural materials. However, since the accuracy of *in silico* methods is not yet perfect, these databases are limited by the fact that the structures of candidates created by their methodologies are different from those derived from real natural products in many cases. Recently, various computer-aided algorithms have been developed for processing high-throughput MS data^28–30^, but those algorithms have mainly focused on primary metabolites, such as sugars, amino acids, and proteins, that were generated by animals.

The primary goal of the present study was to develop LC/MS data-processing protocols that can be easily applied by natural product chemists for the raw MS spectral data of secondary metabolites acquired by the data-independent acquisition (DIA) method. First, the raw MS spectra were processed according to several data-processing steps, such as noise filtering and deisotoping. Then, the data were clustered to obtain representative MS spectra (RMSs) based on a similarity scoring metric between consecutive MS spectra. Next, we used the RMSs to evaluate the chemical diversity in the natural product extracts and discover novel compounds. Using the extracts of *Agrimonia pilosa*, which is native to East Asian countries, as a model, our developed protocols were validated and applied to the discovery of known and novel secondary metabolites. In addition, we attempted to develop a simple method to score the structural novelty of the RMSs in a sample and to build an in-house reference database consisting of natural product metabolites that have not been characterized but exist. We introduced a new scoring system, the Fresh Compound Index (FCI), which evaluates the dissimilarity of the RMSs in a sample against 65,322 reference RMSs obtained from 466 medicinal plants that have been added to our in-house database. This scoring system helps natural product researchers discover unusual secondary metabolites that can contribute to expanding the chemical diversity of natural products.

## Results

### Overview of our LC/MS data-processing protocols for the representative MS spectra

**Figure 1:**
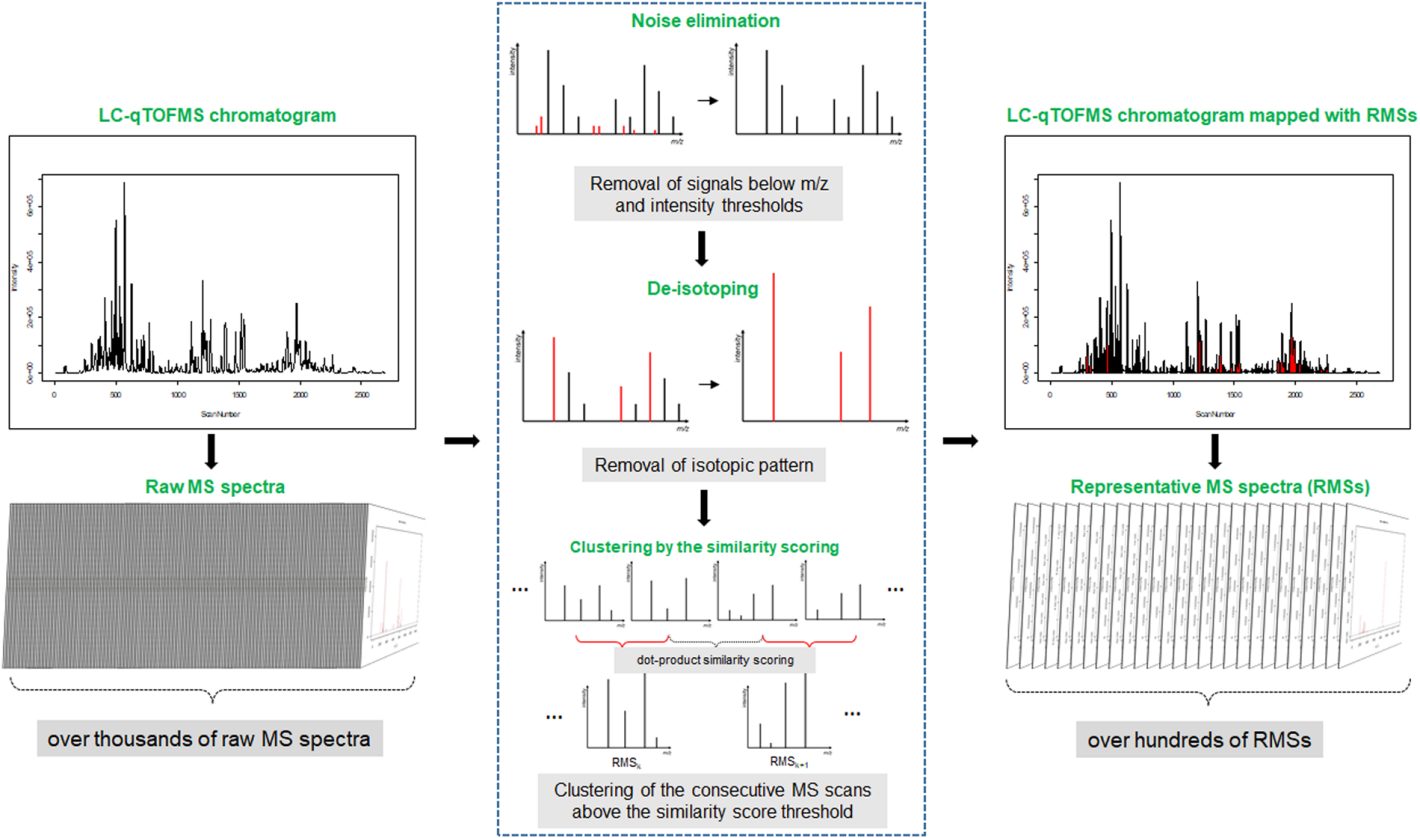
An overview of the acquisition procedure of the RMSs from the raw MS spectra.

We attempted to develop an LC/MS data-processing pipeline to extract the MS spectral information for interpretation of the structures of small secondary metabolites from large quantities of raw MS spectral data. Briefly, the automated protocols developed in this study comprise noise filtering, deisotoping, and clustering after similarity scoring between consecutive MS spectra (Fig. 1). After these automated processes, several thousand raw MS spectral scans from a sample are combined into tens to hundreds of RMSs. The detailed data-processing protocols are presented in Supplementary Note 1. The RMSs are tentatively considered to be derived from single metabolites that are well separated on the UPLC system, and the RMSs are then used to investigate the structural characteristics of the secondary metabolites in the extracts of natural materials.

### Optimization of LC/MS data-processing protocols using model datasets

We compared and optimized the data-processing parameters, including noise filtering, the similarity score thresholds and the deconvolution filters using a natural product extract as a model dataset to improve the quality of the RMSs. The methanolic extracts of *Agrimonia pilosa* (Rosaceae)^31^, which is a perennial plant distributed throughout Korea, Japan and China, were used in the study, along with the spectral information of eight new compounds and six known compounds that have previously been reported from the roots of *A. pilosa*^32^.

**Figure 2:**
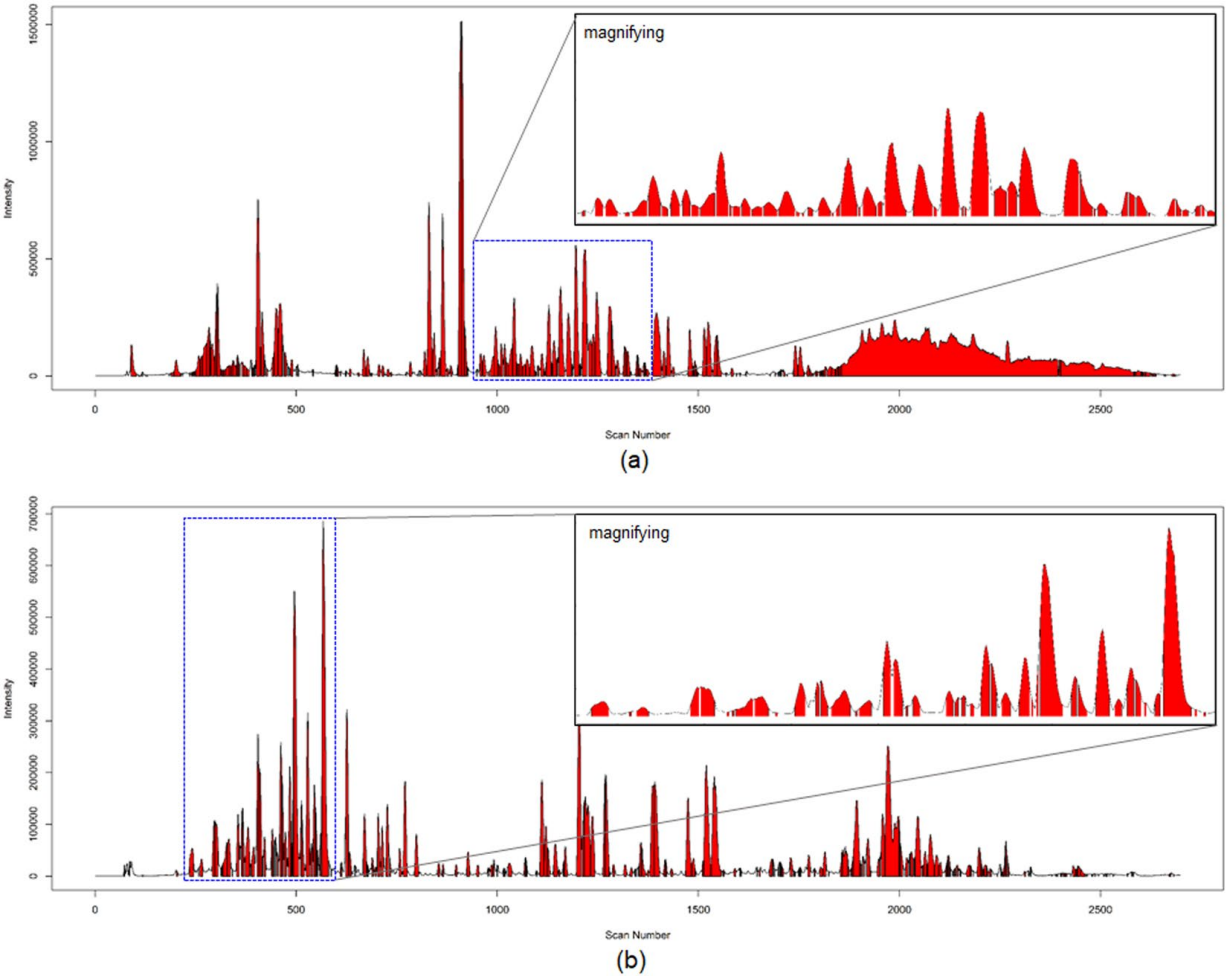
The LC chromatograms of the extracts of *A. pilosa* roots (**a**) and the aerial parts (**b**) RMSs profiles mapped with the RMS profiles colored in red. The similarity score threshold between consecutive MS spectra was set at 0.95.

Our protocols were only focused on the clear separation of the chromatographic peaks based on the acquisition of RMSs. The two raw MS spectral datasets generated from the root and aerial parts of *A. pilosa* were processed and optimized with noise filtering thresholds only for handling the ion peaks with *m/z* and intensity values of over 100 as well as a deisotoping process. The processed MS spectra were clustered between consecutive scans with similarity scores above the threshold using a modified dot-product method to generate the RMSs with noise filtering and deisotoping steps. As the similarity score thresholds were increased to 0.95 (roots) or 0.90 (aerial parts), the number of RMSs gradually increased (Supplementary Fig. S1). Since higher similarity scores, e.g., 0.99, reduced the number of chromatographic peaks apparently derived from single compounds in the samples, the two datasets of raw spectra from the *A. pilosa* roots and aerial parts were processed into 145 and 212 RMSs with similarity scores of 0.95 and 0.90, respectively, which gave separation qualities that were much better than those at higher or lower thresholds (Supplementary Fig. S2). In addition, we applied two deconvolution filters to separate a single RMS into two different spectra when the consecutive MS spectra used to generate a single RMS showed different base peak ions or a convex downward pattern. After clustering based on the similarity scores, two deconvolution filters were applied to separate the unresolved peaks derived from co-eluted compounds (Supplementary Fig. S3). In further studies, we optimized the similarity score threshold to 0.95, which appeared to allow the correct detection of the chromatographic peaks of interest and remove the noise peaks, and two deconvolution filters were used to improve the separation of a single RMS generated from co-eluted metabolites. As a result, two sets of raw MS spectra consisting of 2699 scans were converted to 205 RMSs for roots and 232 for aerial parts (Fig. 2 and Supplementary Table S1).

### Dereplication study of *Agrimonia pilosa*

**Figure 3:**
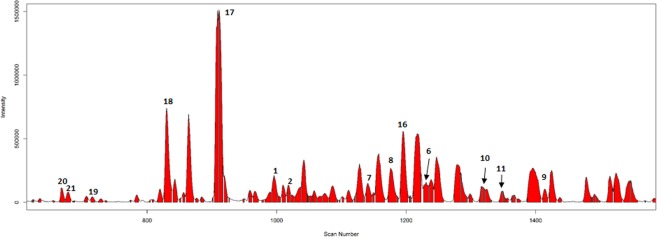
The RMSs profile of fourteen compounds (**1**, **2**, **6**–**11** and **16**–**21**) previously reported from *A. pilosa* roots.

The RMSs corresponding to fourteen compounds (**1**, **2**, **6–11** and **16–21**) that were previously reported successfully were identified in the LC/MS chromatogram of *A. pilosa* roots (Fig. 3 and Supplementary Fig. S4)^31^ and were introduced in the dereplication study to discover other secondary metabolites. The symmetric Pearson’s correlation distance matrix consisting of the similarity score profiles between the RMSs in a sample was applied to the hierarchical clustering analysis (HCA) (Supplementary Fig. S5). We only handled 189 of the 205 total RMSs of *A. pilosa* roots to facilitate the interpretation of the HCA results. The 16 RMSs not applied for the HCA were regarded as the unimportant scans derived from the mixture of nonpolar metabolites, such as lipids, that were highly retained in the column due to their high affinity. The fourteen RMSs for the six agrimonolides (**16–21**) and seven acylphloroglucinolated catechins (**1–2**; pilosanidins, and **7–11**; pilosanols) were grouped with other similar chemical scaffolds in a single sub-cluster on the dendrogram, except for the RMS corresponding to pilosanol A (**6**) (Supplementary Fig. S6).

**Figure 4:**
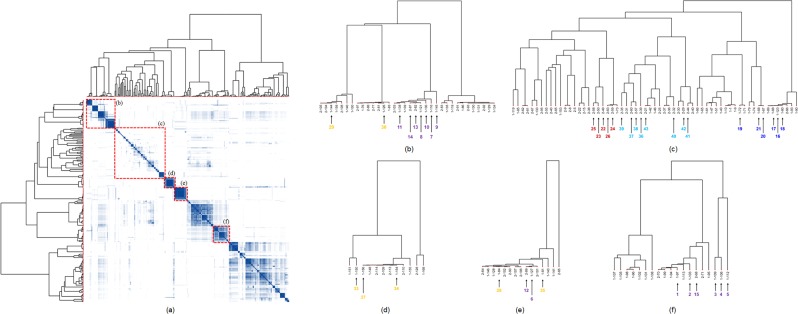
The heatmap of all 236 RMSs from *A. pilosa* (117 for roots and 119 for aerial parts), which each consist of more than four raw spectra (**a**). The HCA was performed with Pearson correlation method as the distance measure and ward.D linkage as the clustering method. The dendrograms for the regions encompassed by the dashed boxes (**b–f**) in the heatmap display the leaves for compounds **1**–**43**, which are shown in violet for pilosanidins (**1**–**5**) and pilosanols (**6**–**15**), blue for agrimolides (**16**–**22**), dark red for chromones (**22**–**26**), yellow for triterpenes (**23**–**35**) and sky blue for flavonoids (**36**–**43**).

Furthermore, the RMSs of the root and aerial parts of *A. pilosa* were used to validate our data-processing protocols and to investigate the unidentified RMSs clustered near the RMSs corresponding to **1**, **2**, **6–11** and **16–21**. Next, 236 RMSs (117 for the roots and 119 for the aerial parts of *A. pilosa*), each consisting of more than four raw spectra, were applied to the HCA together and are shown on the heatmap (Fig. 4). Nine new compounds (**3**–**5**, **12–15**, **25**, and **26**) and twenty-two known compounds (**22–24** and **27–43**) were located around the sub-clusters containing the compounds identified in a previous study (Supplementary Note 2 and 3). Notably, five new compounds, pilosanidin derivatives **3–5** and pilosanols **13** and **14**, which are only found in *A. pilosa*, were found in their expected sub-clusters. Two other new compounds, **25** and **26**, were found in the same sub-cluster as the known chromone derivatives **22–24**, which have the same backbone. Pilosanol-type compound **12** was clustered with **6** in the lowest sub-cluster, which was far from the other pilosanols but near two triterpene derivatives that contain a glucose moiety (**28** and **35**). Additionally, pilosanol **15** was found near the sub-clusters containing pilosanidin derivatives **1**–**5**. The RMSs for nine triterpenes (**27–35**) and eight flavonoids (**36–43**) that were previously reported were tightly clustered according to the MS fragmented patterns that resulted from their backbones or other functionalities, such as the number of oxygen atoms, the presence of double bonds, and the presence of sugar moieties (Supplementary Fig. S7).

### Novelty scoring using the reference RMSs

**Figure 5:**
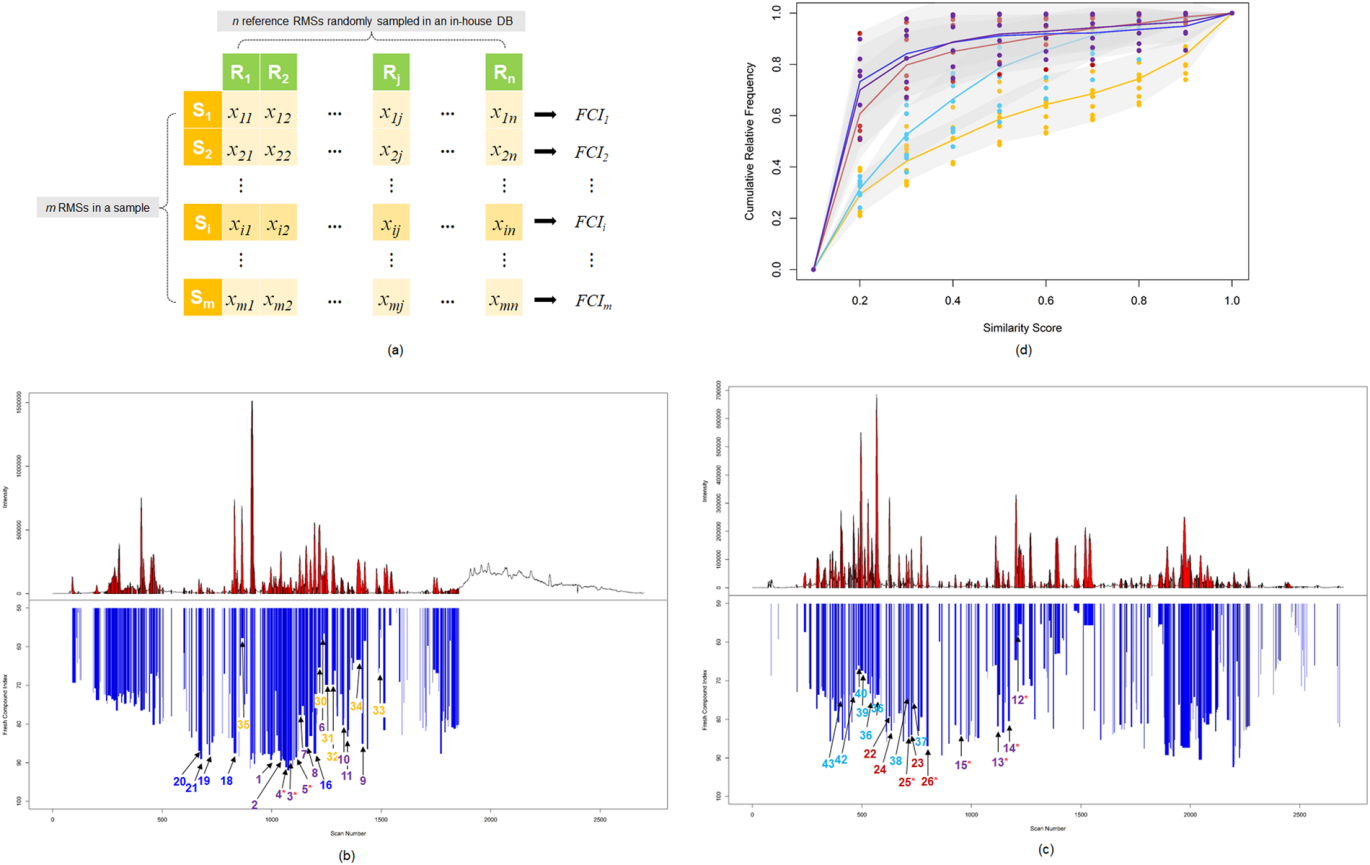
The symmetric matrix consisting of the similarity score profiles between m RMSs in a sample and n reference RMSs in our in-house database for the HCA (**a**). x_i,j_ denotes the dot-product similarity score between the i_th_ RMS (S_i_) in a sample and the j_th_ reference RMS (S_j_) in our in-house database. FCI_i_, the normalized sum of the similarity scores vector of S_i_, represents the structural novelty of a secondary metabolite in a sample relative to the reference RMSs in our in-house database. The LC chromatograms mapped with RMS are shown in red (upper), and the FCIs profile corresponding to the RMSs (lower) from *A. pilosa* roots (**b**) and the aerial parts (**c**). Compounds **1**–**43** are shown in violet for pilosanidins (**1**–**5**) and pilosanols (**6**–**15**), blue for agrimolides (**16**–**22**), dark red for chromones (**22**–**26**), yellow for triterpenes (**23**–**35**) and sky blue for flavonoids (**36**–**43**). The newly isolated compounds (**3**–**5**, **12**–**15**, **25** and **26**) are indicated by a red asterisk. The trend lines of the cumulative relative frequency of the similarity scores of the RMSs corresponding to the chemical scaffolds, pilosanidins and pilosanols (violet), agrimolides (blue), chromones (dark red), triterpenes (yellow) and flavonoids (sky blue) (**d**). The points and solid lines represent the cumulative relative frequency of the similarity scores, which are separated by intervals of 0.1, of each RMS against the reference RMSs in our in-house database and the mean values of the cumulative relative frequency, respectively. The standard deviations of each interval of the points are shaded in gray.

We attempted to devise a method that can identify the MS spectral patterns of secondary metabolites with novel structures, but the exception to this was the common structures, such as triterpenes and flavonoids, that have already been intensively studied or that are produced by many plants. We constructed an in-house database consisting of 65,322 reference RMSs in negative mode using the above user-defined parameters in our LC/MS data-processing protocols. The reference RMSs were derived from metabolites that have not been identified but are unambiguously present in the 466 representative Korean medicinal plants (Supplementary Table S2); some of the representative plants were deposited as standard medicinal herbs in the Korea Plant Extract Bank (Korea Research Institute of Bioscience and Biotechnology, Ministry of Science, ICT and Future Planning, Cheongju, South Korea), and others were directly collected from a Korean medicinal herb garden (Seoul National University, Goyang, South Korea). The garden contained plants that are native to East Asian countries, such as Korea, China and Japan, making these plants the most common sources of medicinal materials for traditional Korean medicines, and their chemical compositions have been intensively studied for many years. We assumed that the 466 plants randomly sampled in Korea are representative of plants native to East Asia, and we used them to construct an in-house database of secondary metabolites to investigate the novelty of RMSs in a given sample. The structural novelty of compounds given by the FCI in a sample was calculated as the normalized value of the dissimilarity and similarity indices against the reference RMSs (Fig. 5a). The RMSs corresponding to the secondary metabolites with more novel structures in *A. pilosa* samples showed higher FCIs than did the RMSs of the metabolites common to many plants (Fig. 5b). The FCIs of pilosanidins (**1–5**), pilosanols (**6**–**15**) and agrimonolides (**16–21**), which are only found in the genus *Agrimonia* or in *A. pilosa*, are 89.4 ± 0.1, 80.4 ± 4.6 and 88.1 ± 0.9, respectively, but the FCIs of triterpenes (**27–35)** or flavonoid derivatives (**36**–**43**), which are common to many plants, are 60.7 ± 7.9 and 72.4 ± 3.6, respectively (Table 1). In addition, the trend lines of the cumulative relative frequency of the similarity scores of the RMSs corresponding to 43 secondary metabolites isolated from *A. pilosa* samples against the reference RMSs in our in-house database indicated patterns similar to the results of the FCI profiles from the different cumulative patterns, which is consistent with the chemical scaffolds (Fig. 5c). The RMSs of triterpenes and flavonoids have relatively higher similarity scores against the reference RMSs than do the scores of the pilosanidins (**1–5**), pilosanols (**6**–**15**), agrimonolides (**16–21**) and chromones (**22**–**25**).

**Table 1:** Secondary metabolites **1**–**43** isolated from *A. pilosa* roots and aerial parts.

No.^a^	Name	Exact molecular weight	Molecular Formula	FCI^b^
**1**	pilosanidin A	814.2472	C_43_H_42_O_16_	89.3
**2**	pilosanidin B	814.2472	C_43_H_42_O_16_	89.5
**3**	pilosanidin C	828.2629	C_44_H_44_O_16_	89.4
**4**	pilosanidin D	798.2523	C_43_H_42_O_15_	89.4
**5**	pilosanidin E	798.2523	C_43_H_42_O_15_	89.2
**6**	pilosanol A	540.1995	C_29_H_32_O_10_	58.4
**7**	pilosanol B	526.1829	C_28_H_30_O_10_	77.7
**8**	pilosanol C	526.1829	C_28_H_30_O_10_	83.1
**9**	isopilosanol A	540.1995	C_29_H_32_O_10_	85.1
**10**	isopilosanol B	526.1829	C_28_H_30_O_10_	80.3
**11**	isopilosanol C	526.1829	C_28_H_30_O_10_	83.3
**12**	epipilosanol A	540.1995	C_29_H_32_O_10_	58.3
**13**	epipilosanol B	526.1829	C_28_H_30_O_10_	81.8
**14**	epipilosanol C	526.1829	C_28_H_30_O_10_	71.5
**15**	epipilosanol N	498.1526	C_26_H_26_O_10_	84.0
**16**	agrimonolide	314.1154	C_18_H_18_O_5_	87.7
**17**	agrimonolide-6-O-Glc^c^	476.1682	C_24_H_28_O_10_	89.2
**18**	agrimonolide-6-O-Araf^d^−6-O-Glc	608.2105	C_29_H_36_O_14_	87.5
**19**	4-hydroxyagrimonolide 6-O-Glc	492.1632	C_24_H_28_O_11_	88.0
**20**	desmethylagrimonolide 6-O-Glc	462.1526	C_23_H_26_O_10_	86.9
**21**	desmethylagrimonolide 4′-O-Glc	462.1526	C_23_H_26_O_10_	89.1
**22**	5,7-dihydroxy-2-propylchromone 7-O-Glc	382.1264	C_18_H_22_O_9_	82.9
**23**	5,7-dihydroxy-2-(1-methylethyl) chromone 7-O-Glc	382.1264	C_18_H_22_O_9_	79.3
**24**	5,7-dihydroxy-2-(1-methylpropyl) chromone 7-O-Glc	396.1420	C_19_H_24_O_9_	84.0
**25**	5,7-dihydroxy-2-(1-methylethyl)chromone-7-O-[6′′-HMG^e^]-*β*-D-glucopyranoside	526.1686	C_24_H_30_O_13_	84.6
**26**	5,7-dihydroxy-2-(1-methylpropyl)chromone-7-O-[6′′-HMG]-*β*-D-glucopyranoside	540.1843	C_25_H_32_O_13_	86.9
**27**	corosolic acid	472.3552	C_30_H_48_O_4_	54.5
**28**	rosamultin	650.4030	C_36_H_58_O_10_	57.8
**29**	2-oxopomolic acid	486.3345	C_30_H_46_O_5_	63.4
**30**	(1*β*,2*α*,3*β*)-1,2,3,19-tetrahydroxyurs-12-en-28-oic acid	504.3451	C_30_H_48_O_6_	65.4
**31**	(1*β*,2*α*)-1,2,19-trihydroxy-3-oxo-urs-12-en-28-oic acid	502.3294	C_30_H_46_O_6_	69.8
**32**	1*β*-hydroxy-2-oxopomolic acid	502.3294	C_30_H_46_O_6_	69.8
**33**	fupenzic acid	484.3189	C_30_H_44_O_5_	65.6
**34**	maslinic acid	472.3552	C_30_H_48_O_4_	50.0
**35**	arjunetin	650.4030	C_36_H_58_O_10_	49.8
**36**	kaempferol-3-*O*-Glc	448.1006	C_21_H_20_O_11_	73.3
**37**	*cis*-tiliroside	594.1373	C_30_H_26_O_13_	75.8
**38**	*trans*-tiliroside	594.1373	C_30_H_26_O_13_	75.3
**39**	apigenin-7-*O*-Glc	432.1056	C_21_H_20_O_10_	67.4
**40**	luteolin-7-*O*-Glc	448.1005	C_21_H_20_O_11_	66.0
**41**	apigenin-7-*O*-GlcA^f^	446.0849	C_21_H_18_O_11_	73.7
**42**	dihydrokaempferol-3-*O*-Glc	450.1162	C_21_H_22_O_11_	73.0
**43**	(2S,3S)-glucodistylin	466.1111	C_21_H_22_O_12_	74.5

^a^Compounds **1**, **2**, **6–11** and **16–21** have been reported in a previous study, and **3**–**5**, **12–15** and **22–43** were isolated in the present study.

^b^FCI: fresh compound index; ^c^Glc: *β*-D-glucose; ^d^Ara*f*: *α*-L-arabinofuranose; ^e^HMG: (*S*)3-hydroxy-3-methylglutaroyl, ^f^GlcA: *β*-D-glucuronic acid.

## Discussion

Discovering compounds with structural novelty from natural products has contributed to expanding the known chemical diversity. Accordingly, the development of methodologies for supporting this process have helped to accelerate natural product research. Hence, various MS-based dereplication approaches have been developed to avoid the rediscovery of known compounds from natural materials^27,33–37^. Recently, a popular approach has been molecular networking (MN), which visualizes the connectivity of molecules with similar MS/MS spectral patterns generated by the data-dependent acquisition (DDA) mode; many natural product chemists have applied MN to the discovery of novel compounds by tracking the connections between the nodes from known metabolites and unknown compounds^38–41^.

In the present study, we developed a new data-processing protocol based on MS spectra that were acquired by the DIA method, which potentially permits the simultaneous fragmentation and detection of peaks regardless of the ion abundances^42^. The raw MS^2^ spectra consisting of fragment ions rapidly and continuously detected from precursor ions in the MS^1^ spectra without an ion selection step were acquired in an unbiased and parallel manner by DIA analyses and converted into the RMSs using our data-processing protocol. The RMSs contain the essential MS spectral information corresponding to every secondary metabolite in a sample and are directly mapped on an LC chromatogram. Our protocol can directly verify the separation performance of a chromatographic method by checking the quality of the well-resolved peaks while adjusting the data-processing parameters.

Furthermore, RMSs can be used for applied studies, such as dereplication studies and the rapid discovery of novel compounds based on the structural relationships between the massive volume of secondary metabolites in natural products using computational methods. The HCA of the symmetric matrix consisting of the similarity scores between the RMSs provided more reliable results than did MN visualized only by the similarity of two nodes. When using *A. pilosa* samples as the model datasets, two RMSs sharing the same ion peaks were connected in MN, but clusters of compounds containing the same chemical scaffolds but lacking common fragments were not connected (Supplementary Fig. S8). On the other hand, compounds generating more similar MS spectral patterns were located in adjacent clusters on the dendrogram, and the HCA of the symmetric Pearson correlation matrix provided more useful information for the discovery of novel compounds than that provided by MN (Fig. 4). Our method was successfully applied to identify structurally similar but novel compounds (**3**–**5** and **12**–**15**) in the sub-clusters adjacent to RMSs that were already known. In addition, new compounds with different backbones, namely, six chromones (**16–21**), were identified in sub-clusters that were far-removed from sub-clusters containing known compounds. High-resolution ultra-performance liquid chromatography (UPLC) was used to obtain highly separated peaks corresponding to as many components as possible in a sample by using a long analysis time prior to MS analysis; however, among many secondary metabolites in the sample, a few pilosanols and triterpenes with similar physiochemical properties were co-eluted from the column and simultaneously detected. Among the 43 compounds isolated from *A. pilosa*, the RMSs of three pilosanols (**6**, **12** and **15**) were far located from the sub-clusters containing the other pilosanols. Their RMSs suggested the presence of other derivatives, which were co-eluted from the column; the signals indicative of triterpene or pilosanidin derivatives were more intense (Supplementary Fig. S9).

**Figure 6:**
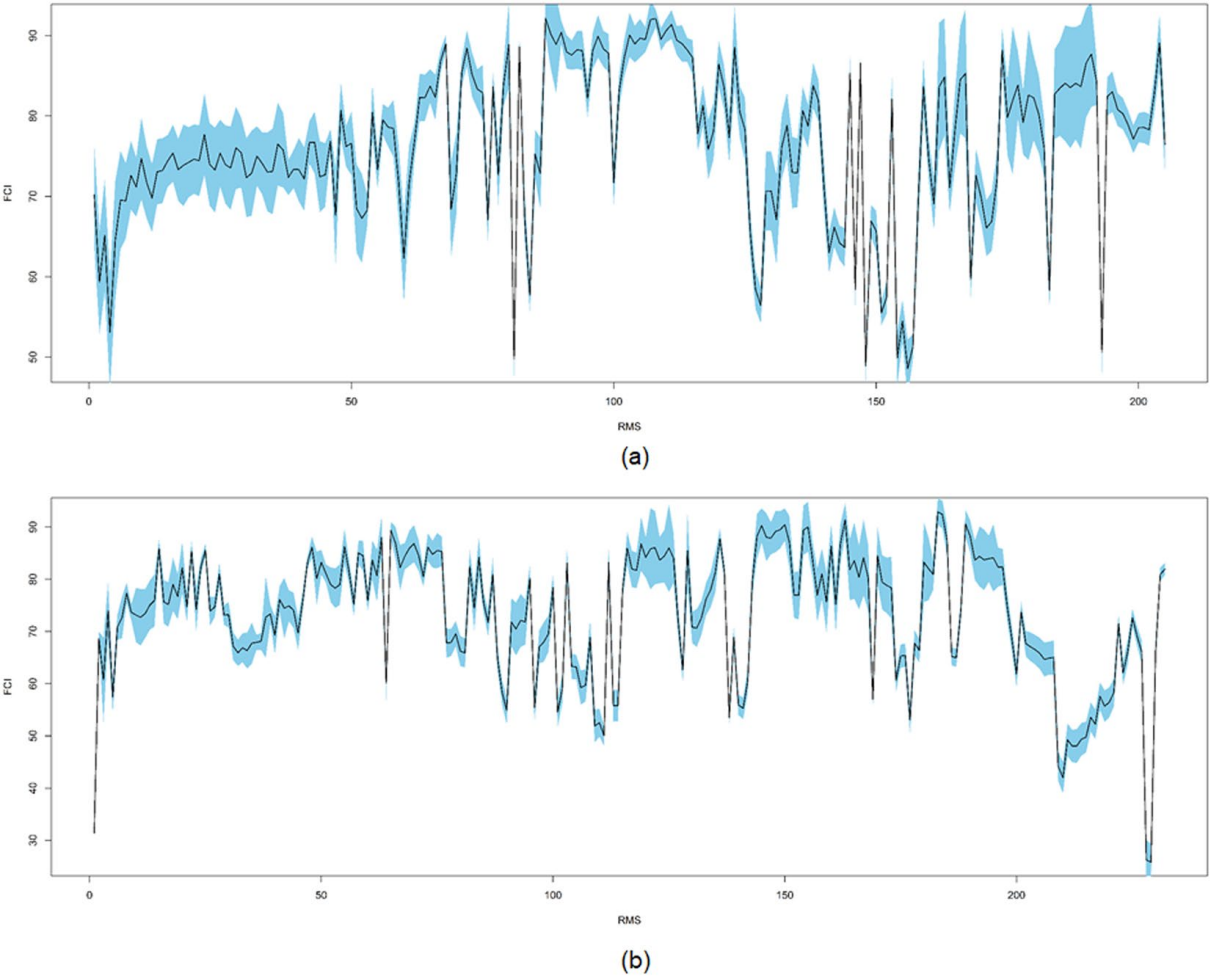
The FCI profiles of the RMSs of *A. pilosa* roots (**a**) and the aerial parts (**b**). The FCIs and the 95% confidence intervals of the FCIs are plotted with solid lines and are shaded in light blue.

In LC/MS metabolomics or dereplication studies, peak identification has focused on finding the exact structures of unknown metabolites in a sample by comparing their spectral data to those of known compounds deposited in mass spectral databases. However, natural product chemists are more interested in the discovery of unknown metabolites that only exist in certain species. In the present study, to discover novel secondary metabolites, we chose to use an MS spectral database that contains unknown secondary metabolites that have not been identified but are unambiguously present in natural products. We introduced a new scoring system, the FCI, which grades the structural novelty of RMSs in a sample against the “real but unknown” reference RMSs in our in-house database. The FCIs of the RMSs in the sample were calculated against the 65,322 reference RMSs from 466 representative Korean medicinal plants, which were automatically extracted by our developed LC/MS data-processing protocols. The 466 samples used to construct our in-house database were regarded as a representative set of the medicinal plants distributed in East Asian countries, including Korea, China and Japan, which have similar climates and geographical conditions. The FCIs can be used for the discovery of secondary metabolites with high structural novelty or with similar chemical scaffolds. Since a 95% confidence interval was selected, the FCI profile with the maximum error in the estimate based on the standard deviations of the FCIs, which were calculated from the reference RMSs in 10 sub-groups with 46–47 species randomly sampled from 466 plants, shows that this new scoring method can be reliably applied to predict the structural novelty of unknown secondary metabolites and to discover new compounds in natural materials (Fig. 6).

The structural complexity of secondary metabolites in natural products is one of the greatest challenges in natural product research. In the present study, we introduced DIA-based LC/MS data-processing protocols that allow natural product chemists to inspect their raw MS data and identify meaningful MS spectral information. In the future, we will continue to add reference RMSs from plants to our in-house database, and we expect that the intensive study of RMSs with higher FCIs will guide the rapid discovery of novel secondary metabolites. This approach will facilitate the laborious and tedious isolation process and accelerate the discovery of novel secondary metabolites.

## Methods

### Plants

*Agrimonia pilosa*, which was used for the optimization of our data-processing protocols, was collected in August 2016 from the Medicinal Plant Garden, Seoul National University, Goyang, Korea, and authenticated by Prof. Tae-Jin Yang (College of Agricultural and Life Sciences, Seoul National University). A voucher specimen of the plant was deposited at the Herbarium of the Medicinal Plant Garden of the College of Pharmacy, Seoul National University. The extracts of 368 Korean medicinal herbs were purchased from the Korea Plant Extract Bank, Korea Research Institute of Bioscience & Biotechnology (KRIBB). An additional 98 plants were collected from the Medicinal Plant Garden, Seoul National University, Goyang, Korea, in August 2016, and their voucher specimens were deposited in the College of Pharmacy, Kangwon National University (Supplementary Table S2). The extracts were dissolved at a concentration of 5 mg/ml in 50 or 100% LC-grade MeOH depending on their solubility. After passage through a 0.2-μm membrane filter (Minisart, Sartorius Stedim Biotech GmbH, Gorttingen, Germany), the samples were stored in a deep freezer at −80 °C.

### Isolation and structural determination of secondary metabolites from *A. pilosa*

The isolations of fourteen secondary metabolites (**1**, **2**, **6–11** and **16–21**) were conducted as previously described in the literature^31^. Twenty-nine compounds (**3–5**, **12**–**15** and **22**–**43**) were isolated from *A. pilosa* roots and aerial parts using a wide range of chromatographic techniques in accordance with the RMSs’ profiles and their FCIs. The structural elucidation of each of these compounds by spectroscopic methods, such as 1D and 2D NMR, MS and UV analyses, is described in detail in Supplementary Note 3.

### UPLC-qTOF analytical conditions

The LC/MS systems consisted of a Waters Acquity UPLC system (Waters Co., Milford, MA, USA) with a binary solvent delivery system and an auto-sampler. The UPLC column was a Waters Acquity UPLC BEH C_18_ (150 mm × 2.1 mm, 1.7 μm). The temperatures of the auto-sampler and the column oven were 15 °C and 40 °C, respectively. The flow rate was 300 μl/min. For the detection of polar and nonpolar metabolites in a sample, the mobile phases were 0.1% formic acid in H_2_O (A) and acetonitrile (B), and the following gradient was used: 5–95% B (0–14 min), 95% B (14–17 min), 50–70% B (10–17 min) and 5% B (17.1–20 min). The injection volume was 2 μl. The MS experiments were performed on a Waters Xevo G2 QTOF mass spectrometer (Waters MS Technologies, Manchester, UK) equipped with an electrospray ionization (ESI) interface. The MS/MS ion patterns were obtained using a collision energy ramp from 15 to 45 eV in MS^E^ mode. The ESI parameters were set as follows: in negative ion mode, a capillary voltage of 2.5 kV, cone voltage of 45 V, source temperature of 120 °C, desolvation temperature of 350 °C, cone gas flow of 50 l/h, and desolvation gas flow of 800 l/h. The ion acquisition rate was 0.25 s with resolution in excess of 20,000 FWHM, and the inter-scan delay time was 0.014 s. The energy for collision-induced dissociation (CID) was set to 4 V for the precursor ion. The mass range was from *m/z* 100 to 1800. The instrument was calibrated using a sodium formate solution as the calibration standard as suggested by the manufacturer, and this calibration allowed for mass accuracies of <5 ppm. To ensure the mass accuracy and reproducibility of the optimized MS conditions, leucine encephalin (*m/z* 554.2615 in negative mode) was used as the reference lock mass at a concentration of 200 pg/μl and a flow rate of 5 μl/min and was sprayed into the MS instrument every 10 s.

### Data processing for the acquisition of RMS

MS spectral data acquired from the UPLC-qTOF instrument were processed by the source codes, which were written in R statistical language (ver. 3.2.2) and are available from the authors upon request. The detailed processing procedures are described in Supplementary Note 1. Briefly, after converting the raw data files into mzXML files, every MS scan in a sample was processed according to the data-processing protocols, such as the removal of higher signal-to-noise signals and the deisotoping step for the monoisotopic patterns. Then, the sum of all the peaks in a processed MS scan was scaled to 1000 to minimize the influence of peaks with high intensities in the similarity scoring step between the consecutive scans. The consecutive processed MS scans with above a user-defined threshold based on a modified dot-product similarity scoring method were combined into an RMS^43^.

### Hierarchical clustering analysis and network visualization of RMSs

For n RMSs, the similarity score of every RMS was calculated by a modified dot-product method against other RMSs in the same sample, and the spectra were compiled into an n × n matrix. The similarity score vectors of each row were hierarchically compared based on several distance methods, such as Euclidean and Pearson, and linkage methods, such as average, centroid, and ward.D, using the ‘Dist’ function of the ‘amap’ package in R. The differences in the sub-clustering of RMSs due to the distance and linkage methods were evaluated based on the dendrograms visualized by the ‘dendlist’ function of the ‘dendextend’ package.

### Calculation and statistical analysis of the FCI values

The general idea of the novelty of the RMSs in a sample, or the FCI, is as follows: the FCI of the *i*_th_ RMS is determined by the difference of two values, the dissimilarity index (DI) and the similarity index (SI). The DI of the *i*_th_ RMS is the ratio of reference RMSs with similarity scores of 0 against the total reference RMSs, and the SI is the weighted sum of the similarity scores against references RMSs with non-zero similarity scores of the total reference RMSs. The FCI is calculated from the following equation:

1 $$FC{I}_{i}={\rm{DI}}-{\rm{SI}}=(\frac{N-m}{N}-\sqrt{\frac{1}{\,m}\times {\sum }_{j=1}^{m}{x}_{ij}^{2}})\times 100\,{\rm{for}}\,i=1,\,2,\,3,\,\cdots ,n$$

where *m* and *X*_*i*_ = {*x*_*i1*_, *x*_*i2*_, $\cdots$, *x*_*ij*_, $\cdots$, *x*_*im*_} denote the number of reference RMSs with non-zero similarity scores against the *i*_th_ RMS among *N* (=65,322) total reference RMSs and the similarity score vector of the *i*_th_ RMS, respectively.

To calculate the 95% confidence intervals for the population mean of the FCIs of the RMSs, the means and standard deviations were repeatedly obtained from 10 groups divided by random sampling without replacement among 466 plants, which are approximately derived from all the medicinal plants in East Asia and processed using the t-distribution. The mean and the 95% confidence intervals were visualized by the solid line and the shaded sky-blue colour by the ‘plot’ function in R.

## Supplementary information

Supplementary Info

## Data Availability

The spectral data used in this study are available from the corresponding author upon request.
