# Supplementary material for: Exploring novel secondary metabolites from natural products using pre-processed mass spectral data
Source: Sci Rep. 2019 Nov 22;9:17430. doi: 10.1038/s41598-019-54078-1 (PMC6874550; doi:10.1038/s41598-019-54078-1)
Supplement: Supplementary file 1 — Supplementary Info [file 41598_2019_54078_MOESM1_ESM.docx]

**Exploring novel secondary metabolites from natural products using pre-processed mass spectral data**

Hyun Woo Kim,^1^ Seong Yeon Choi,^2^ Hyeon Seok Jang,^2^ Byeol Ryu,^1^ Sang Hyun Sung,^1^ Heejung Yang^2,*^

^1^College of Pharmacy and Research Institute of Pharmaceutical Sciences, Seoul National University, Seoul 08826, Korea

^2^Laboratory of Natural Products Chemistry, College of Pharmacy, Kangwon National University, Chuncheon 24341, Korea

* Corresponding authors at:

Laboratory of Natural Products Chemistry, College of Pharmacy, Kangwon National University, Chuncheon 200-701, South Korea. E-mail address: heejyang@kangwon.ac.kr (H. Yang).

**Supplementary Note 1: Data-processing procedures for the representative MS spectra**

**1. Converting raw files:** We developed the data preprocessing pipeline for extracting of the representative spectral information applicable to the further studies. First, the raw vendor-format data were converted into the general data format, mzXML, for processing of massive spectral data using in-house R codes. Besides, these data preprocessing protocols using mzXML format can support the raw data files from major mass spectrometer vendors, such as Waters, Agilent Technologies, AB Sciex, Thermo Fisher Scientific, Bruker Daltonics. In the study, all the experiments were performed on a Waters Xevo G2 system equipped with the quadruple time-of-flight (qTOF) analyzer capable of the acquisition of high-resolution masses. MS^E^ mode, which is data independent analysis (DIA) method to acquire MS1 and MS2 mass spectra in an unbiased and parallel manner, was engaged to harvest the pattern information of molecular and fragment ions from complex components^1^. This technique provided exact mass data of the parent ion for every detectable compound, as well as fragmented ion peaks for its sub-structure. The fragmented ion pattern of single compound provided more rich clues to determine the similarity or dissimilarity between the consecutive MS spectra and was reliably engaged to cluster themselves above a threshold value.

**2. Noise filtering:** After converting raw data files into mzXML ones, background noises in each spectrum were removed to improve data processing efficiency in high-throughput environment and to reduce the possibility of error in the similarity evaluation between spectra. The *m/z* and intensity thresholds could be manually adjusted depending on the experimental conditions, such as *m/z* range of interest, the concentration of a sample injected into MS spectrometer and the performance of ionization for compounds in a sample, respectively in **Supplementary** **Fig. S10**.

**3. Monoisotoping:** Next step is the reduction of the isotopic patterns to monoisotopic peak. The monoisotoping step was summarized in **Supplementary** **Fig. S11**. Though the isotopic patterns are much more useful in determining of molecular formula compared to the monoisotopic peak, they can hinder the rapid computation process of high-throughput data, as well as the electronic or chemical noisy signals^2^. Since secondary molecules in plants are usually singly charged unlike large ones, such as proteins and peptides, which are multiply charged in ESI-MS, we only took care of signals with the charge state *z* = 1^3^. The MS spectrum was scanned from low to high *m/z* values and the mass peaks within 1.0033 ± 0.005 Da, the m/z difference between the isotopes, were only extracted as the isotopic patterns^4,5^. Subsequently, the isotopic patterns were divided into two groups according to the intensity profiles of peaks. For an isotopic pattern with only decreasing intensities, first *m/z* with the strongest intensity and the sum of intensities were regarded as *m/z* and its intensity corresponding to the monoisotopic peak, respectively^6^. For a peak profile showing a jagged pattern due to overlapped peaks derived by more than two ions, the theoretical isotope profile were calculated using 813,997 elemental formula with C, H, O, N, P and S deposited in Pubchem database (April, 2017),^7^ and was subtracted from an isotopic pattern, which were de-isotoped by first *m/z* value and the sum of their intensities into the monoisotopic singly charged signals, and stored in the processed spectrum. It was repeated until no ion peaks remained in a cluster^8^. Consequently, isotope patterns in a spectrum were simplified to monoisotopic peak patterns.

**4. Clustering between the processed MS spectra with monoisotopic peak patterns:** Even trace metabolites were usually released at short time interval and could be detected in at least more than two MS spectra, of which one was usually measured every seconds, during the chromatographic separation. For clustering together of MS spectra derived from the same metabolites, the MS spectra processed in the above steps including of noise filtering and monoisotoping were combined as follows. First, the MS spectra exceeding the threshold value (hereafter the similarity score) were regarded as the spectral data derived from the same metabolites, and were combined into a cluster (**Fig. 1**). In our study, the dot-product similarity scoring method was used to evaluate the similarity between MS spectra, and showed better quality than other equations, Pearson and Spearman coefficients, and Euclidean similarity^3,9^. Next, every MS clusters were deconvoluted by the base peak ions of every MS spectra in a cluster to resolve non-symmetric peaks by the co-eluted metabolites not separated in the column. A MS cluster was separated into different clusters when the base peak ions of the consecutive MS spectra in a MS cluster were different (BPI filter) or showed a convex downward pattern (the chromatographic peak shape filter) (**Supplementary** **Fig. S3**). After these steps, a MS cluster was regarded as the representative MS spectrum derived from a single compound (hereafter the representative MS spectrum, RMS). Consequently, more than thousands of raw MS spectra from a sample can be simplified into a few hundreds of the representative MS spectra, RMSs, with the essential structural information of metabolites.

**Supplementary Note 2: Isolation of the secondary metabolites from *A. pilosa***

Dried whole parts of *A. pilosa* (2.8 kg) were divided into the aerials (1.5 kg) and roots (1.3 kg) and extracted three times with MeOH (4 L × 3) with ultrasonication at 40 °C. The methanol extracts of each part were concentrated using a rotary evaporator to give a crude extract. The methanol extract of aerial parts (164 g) was suspended in H_2_O and portioned successively with *n*-hexane (5 g), CH­_2_Cl_2_ (16 g)_,_ EtOAc (12 g), and *n*-BuOH (20 g) fractions, respectively. The ethylacetate extract was subjected to normal phase MPLC (CH_2_Cl_2_:MeOH 100:0→0:100) and nine fractions (AA to AI) were obtained. The fraction AC was separated using reverse phase MPLC (H_2_O:MeCN 90:10→10:90) to yield subfractions AC1 to AC4. The subfractions AC2 to AC4 were purified by semi-preparative HPLC (isocratic, 45 % AcCN) each other and the compounds **12** to **15** were isolated. The fraction AF was eluted by Sephadex LH 20 (100% MeOH) and subfraction AF1 and AF2 were obtained. The subfraction AF1 was subjected to semipreparative HPLC (isocratic, 30% MeCN) and compounds **22** to **24** were purified. The subfraction AF2 was also separated by using semipreparative HPLC (isocratic, 20% MeCN) and then, compounds **37** and **38** were gained. The fraction AG was separated by reverse phase MPLC (H_2_O:MeCN 90:10→10:90) and divided into 5 subfractions (AG1 to AG5). The subfraction AG1 was subjected to semipreparative HPLC (isocratic, 30% MeCN) and compounds **25** and **26** were isolated. The subfraction AG4 was separated using semipreparative HPLC(isocratic, 35% MeCN) and compound **42** was isolated from AG4. The subfraction AG5 was also separated to yield a compound **36** by semipreparative HPLC (isocratic, 45% MeCN). The subfraction AH was divided into 5 subfractions (AH1 to AH5) by using reverse phase MPLC. Among them, the subfraction AH5 was subjected to semipreparative HPLC (isocratic, 35% MeCN) and compounds **39, 40** and **43**. The subfraction AI was directly subjected to preparative HPLC (isocratic, 35% MeCN) and compound **41** was obtained.

The methanol extract of root parts (39 g) was suspended in H_2_O and partitioned with *n*-hexane, CHCl_3_, and EtOAc fractions respectively. The ethylacetate extract was subjected to normal phase flash column chromatography (CHCl_3_:MeOH 50:1→10:1) and six subfractions (RA to RF) were obtained. The subfraction RA was subjected to normal phase flash column chromatography (CH_2_Cl_2_:MeOH 99:1→0:100) and three subfractions (RA1 to RA3) were obtained. Each subfractions were separated using semipreparative HPLC eluted with 55% MeCN. Finally, compounds **27** to **29** and **31** to **34** were purified. The subfraction RB was separated by Sephadex LH 20 eluted with 100% methanol and two subfraction, RB1 and RB2 were gained. The subfraction RC was divided into two subfractions, RC1 and RC2 by Sephadex LH 20 size exclusion chromatography (100% MeOH). The subfraction RC1 was subjected to semipreparative HPLC (isocratic, 45 % MeCN). And then compound **30** was isolated. The subfraction RD was eluted with 100% MeOH on Sephadex LH 20 chromatography to yield two subfractions, RD1 and RD2. The RD1 was subjected to semipreparative HPLC (isocratic, 45% MeCN) and compound **35** was obtained. The subfraction RF was divided into four subfractions (RF1 to RF4). The subfraction F4 was separated using semipreparative HPLC (isocratic, 45% MeCN) to yield compounds **3** to **5**.

**Supplementary Note 3: Structural determination of the secondary metabolites (18-37) for *A. pilosa***

Compound **3** was isolated as a brown solid. The molecular formula of **3**, C_44_H_44_O_16_ was determined from the HRESIMS, which showed a deprotonated molecular ion at *m*/*z* 827.2547 [M - H]^-^. Analysis of ^1^H and ^13^C NMR spectra confirmed that compound **3** is a pilosanidin derivative which was reported from our previous study. Two AMX spin system in aromatic proton region (*δ*_H_ 5.6 – 7.0), two methine proton signals at *δ*_H_ 4.95 (1H, d, *J* = 5.3 Hz, H-2t) and 4.42 (1H, d, *J* = 10.1 Hz, H-2u) and two oxygenated carbon signals at *δ*_c_ 85.8 (C-2u) and 81.0 (C-2t) showed the presence of two catechins units. From the coupling constants of the two methine proton signals (H-2u, H-2t), two catechin units were inferred as catechin and epi-catechin respectively. The HMBC correlation signal between *δ*_H_ 4.48 (1H, d, *J* = 7.0 Hz, H-4u) and *δ*_c_ 108.0 (C-8t) indicated that **3** has procyanidin B type (C4 → C8) interflavan linkage. The ^1^H NMR spectrum showed resonances for one methoxy group at *δ*_H_ 3.60 (3H, s, O-CH­_3_), one arylmethyl group at *δ*_H_ 1.90 (3H, s, H-8′′u) and one aliphatic group at *δ*_H_ 3.54 (1H, m, H-10′′), 1.71 (1H, m, H-11′′a), 1.36 (1H, m, H-11′′b), 0.85 (3H, t, *J* = 7.5, H-12′′u) and 1.14 (3H, d, *J* = 6.7, H-13′′u) suggested the presence of an acylphloroglucinol moiety. From the analysis of ^1^H-^1^H COSY spectrum, the acyl group was identified as a 2-methylbutanoyl group. The linkage of procyanidin and acylphloroglucinol units were determined by analysis of the HMBC correlation. The HMBC correlation of methylene protons at *δ*_H_ 3.62 (1H, d, *J* = 15.6, H-7′′u a) and 3.53 (1H, d, *J* = 15.6, H-7′′u b) with aromatic quaternary carbons at *δ*_c_ 106.0 (C-8u) and *δ*_c_ 110.9 (C-1′′u) signified acylphloroglucinol moiety was substituted at C-8u via C-7′′ methylene group. The absolute configuration of the interflavan linkage (C4 → C8) was determined by analysis of ECD spectrum. The Cotton effect at 220 – 240 nm imply the orientation of the 4-flavanyl linkage. In the ECD spectrum of **3**, a strong negative Cotton effect was seen at 220 – 240 nm which meant the configuration of C-4 to be *R*. From these results, compound **3** was named pilosanidin C.

Compound **4** was isolated as a brownish amorphous solid, for which the molecular formula, C­_43_H_42_O_15_ was deduced by HRESIMS data. Two methyl proton signals at *δ*_H_ 1.13 (3H, d, *J* = 6.7, H-11′′u) and 1.09 (3H, d, *J* = 6.7, H-11′′u) and one methine protons at *δ*_H_ 3.60 (1H, m) signified that **27** had methyl 2-propanoyl group instead of butanoyl group of **5**. The ECD spectrum of **4** showed a negative Cotton effect at 220 – 240 nm, indicating the configuration at C-4 to be *R*. From these results, compound **4** was named pilosanidin D.

Compound **5** was obtained as a brownish, amorphous solid. Its molecular formula was determined to be C_43_H_42_O_15_ by analysis of HRESIMS (*m*/*z* 797.2422 [M-H]^-^, calcd for C_43_H_41_O_15_, 797.2455). This formula was same as that of compound **5**, and the ^1^H and ^13^C NMR spectrum of these two compounds were similar. On comparing the ^1^H and ^13^C NMR spectra of **5** with those of pilosanidin B, AA′XX′ aromatic spin system at *δ*_H_ 7.30 (2H, d, *J* = 8.6 Hz, H-2′u) and 6.82 (2H, d, *J* = 8.6 Hz, H-3′u) suggested that upper unit of **5** was an afzelechin instead of a catechin. The analysis of ^1^H-^1^H COSY spectrum announced that the acyl group of phloroglucinol moiety was a butanoyl group. The HMBC correlations of methylene protons at *δ*_H_ 3.62 (1H, d, *J* = 15.6, H-7′′a) and 3.55 (1H, d, *J* = 15.6, H-7′′b) with aromatic quaternary carbons at *δ*_c_ 111.2 (C-1′′u) and 106.1 (C-8u) suggested that phloroglucinol unit was substituted at C-8u via C-7′′ methylene group. The linkage between a afzelchin and a catechin was also assigned to be a C-4 → C8 interflavan linkage by the analysis of HMBC correlations. The ECD spectrum of **5** showed a negative Cotton effect at 220 – 240 nm, suggesting the absolute configuration of C-4 to be *R*. Consequently, compound **5** was named pilosanidin E.

Compound **12** was isolated as brownish amorphous solid. Molecular formula of **12** was determined to be C_29_H_32_O_10_ by HRESIMS analysis. Its ^1^H NMR spectrum was similar to those of epipilosanol derivatives such as proton signals of epicatechin and phloroglucinol moieties. The COSY correlations of aliphatic proton signals at δ_H_ 3.62 (H-10′′), δ_H_ 1.90 (H-11′′a), δ_H_ 1.44 (H-11′′b), δ_H_ 1.21 (H-13′′), δ_H_ 0.89 (H-12′′) and carbonyl carbon signlas at δ_C_ 210.5 in ^13^C NMR spectrum suggested a methylbutanoyl group of a phloroglucinol unit. From the HMBC correlations of H-7′′ with C-8 and C-1′′, the substituted position of acylphloglucinol unit was confirmed as C-8 via a C-7′′ methylene group. Because of negative Cotton effect observed at 280nm from ECD spectrum of **12,** the absolute configuration of C-2 was deterimined to be 2*R*. From these results, compound **12** was indentifed as epipilosanol A, the epimer of pilosanol A (**6**)^10^.

Compound **13** was brownish amorphous solid. Its molecular formula was determined as C_28_H_30_O_10_ by analysis of HRESIMS spectrum. In a ^1^H NMR spectrum, ABX patterned aromatic proton signals at δ_H_ 7.44 (H-2′), δ_H_ 7.35 (H-3′) and δ_H_ 7.99 (H-6′), one aromatic proton signals at δ_H_ 6.62 (H-6), two oxygenated methine proton signals at δ_H_ 5.45 (H-2), δ_H_ 4.72 (H-3), methylene proton signals at δ_H_ 3.54 (H-4a) and δ_H_ 3.36 (H-4b) showed the catechin unit of **13**. From the analysis of COSY spectrum of **13**, methylpropanoyl group of acylphloroglucinol unit was observed. The substituted position of acylphloroglucinol unit on epicatechin was determined to be C-8 by analysis of HMBC spectrum. The H-7′′ proton signal was correlated with C-8 and C-1′′ on HMBC spectrum. The absolute configuration of C-2 was determined by analysis of ECD spectrum. The negative Cotton effect was observed at 280 nm, so the absolute configuration of C-2 was confirmed as 2*R*. From these results, compound **13** was named epipilosanol B which was epimer of pilosanol B (**7**)^10^.

Compound **14** was brownish amorphous solid. Its molecular formula was determined as C_28_H_30_O_10_ by analysis of HRESIMS spectrum. Comparing the ^1^H NMR spectrum of **14** with that of **13**, almost of signals were same, but the acyl group signals at δ_H_ 1.20 (H-11′′, H-12′′) and δ_H_ 3.69 (H-10′′) were different. From the analysis of COSY spectrum, this acyl group was confirmed as butanoyl group. Because of these observations, compound **14** was suggested to be butanoyl substituted epipilosanol derivatives. The bond between acylphloroglucinol group and catechin group was located at C-8 via C-7′′, which was identified by the HMBC correlation of H-7′′ with C-8 and C-1′′. The absolute configuration of C-2 was deterimined to be 2*R* by analysis of ECD spectrum of **14** which showed negative Cotton effect at 280 nm. From these results, compound **14** was indentifed as epipilosanol C, the epimer of pilosanol C (**8**)^10^.

Compound **15** was brownish amorphous solid. Its molecular formula was determined as C_26_H_26_O_10_ by analysis of HRESIMS spectrum. From the broad singlet proton signal of H-2, this catechin unit identified as epi-catechin. These results were similar to those of **13**, so **15** was suggested to be epipilosanol derivatives. In a ^1^H NMR spectrum, ABX patterned aromatic proton signals at δ_H_ 7.44 (H-2′), δ_H_ 7.35 (H-3′) and δ_H_ 7.98 (H-6′), one aromatic proton signals at δ_H_ 6.66 (H-6), two oxygenated methine proton signals at δ_H_ 5.46 (H-2), δ_H_ 4.72 (H-3), methylene proton signals at δ_H_ 3.54 (H-4a) and δ_H_ 3.37 (H-4b) showed the catechin unit of **15**. From the broad singlet proton signal of H-2, this catechin unit identified as epi-catechin. In ^13^C NMR spectrum, three oxygenated aromatic carbon signals at δ_C_ 163.1 (C-2″), δ_C_ 161.7 (C-4″), and δ_C_ 160.4 (C-6″), three aromatic quaternary carbon signals at δ_C_ 111.6 (C-1″), δ_C_ 111.4 (C-3″), and δ_C_ 111.6 (C-5″), one methylene carbon signal at δ_C_ 17.2 (C-7″), one methoxy carbon signal at δ_C_ 61.7 (OCH_3_-4″), one carbonyl carbon signal at δ_C_ 203.8 (C-9″), and one methyl carbon signal at δ_C_ 31.6 (C-1″) suggested the presence acyl phloroglucinol group. From the HMBC correlations of H-7″ with C-8 and C-1″, the substituted position of acyl phloroglucinol group was located at C-8. The absolute configuration of C-2 was determined by analysis of ECD spectrum. The negative Cotton effect was observed at 280 nm, so the absolute configuration of C-2 was confirmed as 2*R*. From these results, compound **15** was named epipilosanol N which was epimer of pilosanol N^11^.

Compound **25** was purified as colorless amorphous solid, of which the molecular formula was determined to be C_24_H_30_O_13_ by HRESIMS analysis. In the ^1^H NMR spectrum, two meta coupled proton signals at δ_H_ 6.67 (1H, br s, H-8) and 6.48 (1H, br s, H-6), and one singlet olefinic proton signal at δ_H_ 6.12 (1H, s, H-3) was observed, which are also shown in the ^1^H NMR spectrum of 5,7-dihydroxy-2-(1-methylethyl) chromone 7-*O*-*β*-D-glucopyranoside (**23**). In comparison of ^1^H and ^13^C NMR spectra of **25** with those of **23**, most of signals were similar but downfield shifted glucose proton signals of H-6′′ (*δ*_H_ 4.49 and 4.23). Additional four doublet signals at δ_H_ 2.76 (1H, d, *J* = 14.5 Hz, H-2′′′a), 2.69 (1H, d, *J* = 14.5 Hz, H-2′′′b), 2.63 (1H, d, *J* = 15.0 Hz, H-4′′′a), and 2.57 (1H, d, *J* = 15.0 Hz, H-4′′′b) and one methyl signal at 1.32 (3H, s, H-6′′′) suggested the presence of a 3-hydroxy-3-methylglutaroyl (HMG) moiety. The HMBC correlation between a glucosyl proton (H-6′′) and a carbonyl carbon at *δ*_C ­_171.0 (C-1′′′) suggested the 6′′-*O*-ether substitution of the HMG moiety. Consequently, the structure of **25** was determined as 5,7-dihydroxy-2-isopropylchromone-7-*O*-[6′′-(3-hydroxy-3-methylglutaroyl)]-*β*-D-glucopyranoside.

Compound **26** was obtained as colorless amorphous solid. Its molecular formula, C­_25_H_32_O_13_ was deduced by HRESIMS spectrum (*m*/*z* 539.1760 [M-H]^-^, calcd for C_25_H_31_O_13_, 539.1765). Comparison of ^1^H and ^13^C NMR spectra of **26** with those of compound **25** suggested that **26** was also HMG substituted chromone with different aliphatic moiety. From the analysis of ^1^H-^1^H COSY spectrum, the aliphatic moiety was identified as sec butyl group. The HMBC correlation between glucosyl proton (H-6′′) and a carbonyl carbon at *δ*_C_ 172.4 (C-1′′′) suggested the 6′′-*O*-ehter substitution of the HMG moiety. From these results, the structure of **26** was identified as 5,7-dihydroxy-2-(1-methylpropyl)chromone-7-*O*-[6′′-(3-hydroxy-3-methylglutaroyl)]-β-D-glucopyranoside. The absolute configurations of HMG moiety of **25** and **26** were determined to be (*S*) by a converted method including amidation, reduction,a dn acetylation as well as by comparison of the ^1^H NMR data with a reference value^12^.

**Supplementary Tables:**

**Table S1.** RMSs from *A. pilosa* roots and aerial parts

| Scan Information^a^ | Compound Name | RMS name |  | Scan Information | Compound Name | RMS name |
| --- | --- | --- | --- | --- | --- | --- |
| 89 - 104 lth= 16 |  | 1-1 |  | 72 - 74 lth= 3 |  | 2-1 |
| 108 - 110 lth= 3 |  | 1-2 |  | 85 - 86 lth= 2 |  | 2-2 |
| 112 - 113 lth= 2 |  | 1-3 |  | 118 - 119 lth= 2 |  | 2-3 |
| 116 - 118 lth= 3 |  | 1-4 |  | 203 - 205 lth= 3 |  | 2-4 |
| 120 - 121 lth= 2 |  | 1-5 |  | 235 - 245 lth= 11 |  | 2-5 |
| 125 - 126 lth= 2 |  | 1-6 |  | 257 - 258 lth= 2 |  | 2-6 |
| 129 - 130 lth= 2 |  | 1-7 |  | 261 - 268 lth= 8 |  | 2-7 |
| 184 - 185 lth= 2 |  | 1-8 |  | 292 - 297 lth= 6 |  | 2-8 |
| 189 - 196 lth= 8 |  | 1-9 |  | 298 - 306 lth= 9 |  | 2-9 |
| 197 - 212 lth= 16 |  | 1-10 |  | 313 - 314 lth= 2 |  | 2-10 |
| 213 - 214 lth= 2 |  | 1-11 |  | 316 - 317 lth= 2 |  | 2-11 |
| 217 - 218 lth= 2 |  | 1-12 |  | 318 - 328 lth= 11 |  | 2-12 |
| 235 - 236 lth= 2 |  | 1-13 |  | 329 - 337 lth= 9 |  | 2-13 |
| 240 - 242 lth= 3 |  | 1-14 |  | 341 - 342 lth= 2 |  | 2-14 |
| 244 - 247 lth= 4 |  | 1-15 |  | 352 - 358 lth= 7 |  | 2-15 |
| 248 - 249 lth= 2 |  | 1-16 |  | 364 - 366 lth= 3 |  | 2-16 |
| 250 - 261 lth= 12 |  | 1-17 |  | 367 - 371 lth= 5 |  | 2-17 |
| 262 - 266 lth= 5 |  | 1-18 |  | 373 - 384 lth= 12 |  | 2-18 |
| 267 - 277 lth= 11 |  | 1-19 |  | 387 - 388 lth= 2 |  | 2-19 |
| 278 - 285 lth= 8 |  | 1-20 |  | 389 - 396 lth= 8 |  | 2-20 |
| 286 - 288 lth= 3 |  | 1-21 |  | 403 - 407 lth= 5 | **43** | 2-21 |
| 289 - 297 lth= 9 |  | 1-22 |  | 409 - 414 lth= 6 |  | 2-22 |
| 299 - 303 lth= 5 |  | 1-23 |  | 416 - 417 lth= 2 |  | 2-23 |
| 304 - 311 lth= 8 |  | 1-24 |  | 420 - 424 lth= 5 |  | 2-24 |
| 313 - 316 lth= 4 |  | 1-25 |  | 439 - 443 lth= 5 |  | 2-25 |
| 317 - 320 lth= 4 |  | 1-26 |  | 444 - 446 lth= 3 |  | 2-26 |
| 321 - 325 lth= 5 |  | 1-27 |  | 448 - 450 lth= 3 |  | 2-27 |
| 326 - 332 lth= 7 |  | 1-28 |  | 453 - 455 lth= 3 |  | 2-28 |
| 333 - 337 lth= 5 |  | 1-29 |  | 459 - 464 lth= 6 | **42** | 2-29 |
| 338 - 342 lth= 5 |  | 1-30 |  | 465 - 468 lth= 4 |  | 2-30 |
| 343 - 351 lth= 9 |  | 1-31 |  | 471 - 476 lth= 6 |  | 2-31 |
| 352 - 355 lth= 4 |  | 1-32 |  | 481 - 487 lth= 7 | **40** | 2-32 |
| 356 - 359 lth= 4 |  | 1-33 |  | 488 - 489 lth= 2 |  | 2-33 |
| 360 - 361 lth= 2 |  | 1-34 |  | 491 - 492 lth= 2 |  | 2-34 |
| 365 - 369 lth= 5 |  | 1-35 |  | 493 - 506 lth= 14 | **39** | 2-35 |
| 370 - 376 lth= 7 |  | 1-36 |  | 511 - 514 lth= 4 |  | 2-36 |
| 377 - 383 lth= 7 |  | 1-37 |  | 515 - 520 lth= 6 |  | 2-37 |
| 389 - 390 lth= 2 |  | 1-38 |  | 525 - 533 lth= 9 |  | 2-38 |
| 391 - 393 lth= 3 |  | 1-39 |  | 536 - 540 lth= 5 | **36** | 2-39 |
| 394 - 395 lth= 2 |  | 1-40 |  | 543 - 547 lth= 5 |  | 2-40 |
| 396 - 398 lth= 3 |  | 1-41 |  | 548 - 551 lth= 4 |  | 2-41 |
| 401 - 411 lth= 11 |  | 1-42 |  | 553 - 554 lth= 2 |  | 2-42 |
| 413 - 420 lth= 8 |  | 1-43 |  | 559 - 562 lth= 4 |  | 2-43 |
| 422 - 423 lth= 2 |  | 1-44 |  | 564 - 580 lth= 17 | **41** | 2-44 |
| 426 - 429 lth= 4 |  | 1-45 |  | 582 - 583 lth= 2 |  | 2-45 |
| 440 - 444 lth= 5 |  | 1-46 |  | 587 - 588 lth= 2 |  | 2-46 |
| 446 - 454 lth= 9 |  | 1-47 |  | 610 - 612 lth= 3 |  | 2-47 |
| 456 - 465 lth= 10 |  | 1-48 |  | 613 - 614 lth= 2 |  | 2-48 |
| 466 - 467 lth= 2 |  | 1-49 |  | 623 - 630 lth= 8 | **22** | 2-49 |
| 469 - 470 lth= 2 |  | 1-50 |  | 632 - 637 lth= 6 | **24** | 2-50 |
| 471 - 476 lth= 6 |  | 1-51 |  | 646 - 648 lth= 3 |  | 2-51 |
| 479 - 481 lth= 3 |  | 1-52 |  | 667 - 674 lth= 8 |  | 2-52 |
| 484 - 485 lth= 2 |  | 1-53 |  | 676 - 677 lth= 2 |  | 2-53 |
| 487 - 492 lth= 6 |  | 1-54 |  | 678 - 680 lth= 3 |  | 2-54 |
| 499 - 500 lth= 2 |  | 1-55 |  | 688 - 692 lth= 5 |  | 2-55 |
| 503 - 504 lth= 2 |  | 1-56 |  | 696 - 698 lth= 3 |  | 2-56 |
| 505 - 506 lth= 2 |  | 1-57 |  | 702 - 709 lth= 8 | **38** | 2-57 |
| 541 - 543 lth= 3 |  | 1-58 |  | 711 - 717 lth= 7 | **25** | 2-58 |
| 597 - 598 lth= 2 |  | 1-59 |  | 723 - 730 lth= 8 | **23** | 2-59 |
| 599 - 601 lth= 3 |  | 1-60 |  | 732 - 735 lth= 4 | **37** | 2-60 |
| 602 - 605 lth= 4 |  | 1-61 |  | 756 - 762 lth= 7 |  | 2-61 |
| 610 - 611 lth= 2 |  | 1-62 |  | 766 - 776 lth= 11 |  | 2-62 |
| 622 - 623 lth= 2 |  | 1-63 |  | 795 - 804 lth= 10 | **26** | 2-63 |
| 624 - 625 lth= 2 |  | 1-64 |  | 852 - 857 lth= 6 |  | 2-64 |
| 632 - 636 lth= 5 |  | 1-65 |  | 863 - 867 lth= 5 |  | 2-65 |
| 654 - 657 lth= 4 |  | 1-66 |  | 896 - 900 lth= 5 |  | 2-66 |
| 665 - 673 lth= 9 | **20** | 1-67 |  | 924 - 931 lth= 8 |  | 2-67 |
| 674 - 682 lth= 9 | **21** | 1-68 |  | 949 - 954 lth= 6 | **15** | 2-68 |
| 685 - 686 lth= 2 |  | 1-69 |  | 977 - 979 lth= 3 |  | 2-69 |
| 687 - 688 lth= 2 |  | 1-70 |  | 984 - 988 lth= 5 |  | 2-70 |
| 703 - 708 lth= 6 |  | 1-71 |  | 990 - 993 lth= 4 |  | 2-71 |
| 713 - 719 lth= 7 | **19** | 1-72 |  | 999 - 1001 lth= 3 |  | 2-72 |
| 725 - 732 lth= 8 |  | 1-73 |  | 1015 - 1016 lth= 2 |  | 2-73 |
| 735 - 738 lth= 4 |  | 1-74 |  | 1017 - 1019 lth= 3 |  | 2-74 |
| 774 - 775 lth= 2 |  | 1-75 |  | 1023 - 1024 lth= 2 |  | 2-75 |
| 781 - 787 lth= 7 |  | 1-76 |  | 1027 - 1036 lth= 10 |  | 2-76 |
| 803 - 805 lth= 3 |  | 1-77 |  | 1067 - 1070 lth= 4 |  | 2-77 |
| 808 - 810 lth= 3 |  | 1-78 |  | 1071 - 1072 lth= 2 |  | 2-78 |
| 816 - 824 lth= 9 |  | 1-79 |  | 1073 - 1074 lth= 2 |  | 2-79 |
| 826 - 838 lth= 13 | **18** | 1-80 |  | 1097 - 1099 lth= 3 |  | 2-80 |
| 840 - 847 lth= 8 | **35** | 1-81 |  | 1106 - 1116 lth= 11 |  | 2-81 |
| 850 - 852 lth= 3 |  | 1-82 |  | 1117 - 1124 lth= 8 | **13** | 2-82 |
| 855 - 858 lth= 4 |  | 1-83 |  | 1125 - 1131 lth= 7 |  | 2-83 |
| 860 - 872 lth= 13 | **28** | 1-84 |  | 1140 - 1150 lth= 11 |  | 2-84 |
| 875 - 879 lth= 5 |  | 1-85 |  | 1153 - 1156 lth= 4 |  | 2-85 |
| 883 - 888 lth= 6 |  | 1-86 |  | 1164 - 1166 lth= 3 | **14** | 2-86 |
| 901 - 902 lth= 2 |  | 1-87 |  | 1167 - 1175 lth= 9 |  | 2-87 |
| 904 - 910 lth= 7 | **17** | 1-88 |  | 1197 - 1209 lth= 13 |  | 2-88 |
| 911 - 917 lth= 7 |  | 1-89 |  | 1211 - 1216 lth= 6 | **12** | 2-89 |
| 919 - 924 lth= 6 |  | 1-90 |  | 1217 - 1231 lth= 15 |  | 2-90 |
| 926 - 927 lth= 2 |  | 1-91 |  | 1233 - 1242 lth= 10 |  | 2-91 |
| 948 - 950 lth= 3 |  | 1-92 |  | 1263 - 1268 lth= 6 |  | 2-92 |
| 957 - 962 lth= 6 |  | 1-93 |  | 1269 - 1278 lth= 10 |  | 2-93 |
| 964 - 972 lth= 9 |  | 1-94 |  | 1279 - 1280 lth= 2 |  | 2-94 |
| 977 - 980 lth= 4 |  | 1-95 |  | 1287 - 1292 lth= 6 |  | 2-95 |
| 982 - 992 lth= 11 |  | 1-96 |  | 1315 - 1321 lth= 7 |  | 2-96 |
| 993 - 1001 lth= 9 | **1** | 1-97 |  | 1330 - 1336 lth= 7 |  | 2-97 |
| 1002 - 1006 lth= 5 |  | 1-98 |  | 1342 - 1344 lth= 3 |  | 2-98 |
| 1008 - 1014 lth= 7 |  | 1-99 |  | 1345 - 1346 lth= 2 |  | 2-99 |
| 1016 - 1021 lth= 6 |  | 1-100 |  | 1350 - 1352 lth= 3 |  | 2-100 |
| 1022 - 1027 lth= 6 |  | 1-101 |  | 1354 - 1359 lth= 6 |  | 2-101 |
| 1028 - 1038 lth= 11 |  | 1-102 |  | 1360 - 1368 lth= 9 |  | 2-102 |
| 1039 - 1047 lth= 9 | **2** | 1-103 |  | 1373 - 1377 lth= 5 |  | 2-103 |
| 1048 - 1049 lth= 2 |  | 1-104 |  | 1380 - 1389 lth= 10 |  | 2-104 |
| 1050 - 1055 lth= 6 |  | 1-105 |  | 1390 - 1404 lth= 15 |  | 2-105 |
| 1056 - 1062 lth= 7 | **5** | 1-106 |  | 1414 - 1418 lth= 5 |  | 2-106 |
| 1063 - 1070 lth= 8 |  | 1-107 |  | 1419 - 1422 lth= 4 |  | 2-107 |
| 1071 - 1080 lth= 10 |  | 1-108 |  | 1432 - 1436 lth= 5 |  | 2-108 |
| 1081 - 1092 lth= 12 | **3** | 1-109 |  | 1468 - 1483 lth= 16 |  | 2-109 |
| 1093 - 1095 lth= 3 |  | 1-110 |  | 1484 - 1492 lth= 9 |  | 2-110 |
| 1097 - 1098 lth= 2 |  | 1-111 |  | 1493 - 1494 lth= 2 |  | 2-111 |
| 1101 - 1104 lth= 4 | **4** | 1-112 |  | 1510 - 1512 lth= 3 |  | 2-112 |
| 1108 - 1116 lth= 9 |  | 1-113 |  | 1514 - 1531 lth= 18 |  | 2-113 |
| 1117 - 1118 lth= 2 |  | 1-114 |  | 1532 - 1557 lth= 26 |  | 2-114 |
| 1120 - 1122 lth= 3 |  | 1-115 |  | 1561 - 1564 lth= 4 |  | 2-115 |
| 1123 - 1134 lth= 12 | **7** | 1-116 |  | 1589 - 1593 lth= 5 |  | 2-116 |
| 1135 - 1136 lth= 2 |  | 1-117 |  | 1603 - 1604 lth= 2 |  | 2-117 |
| 1138 - 1147 lth= 10 |  | 1-118 |  | 1606 - 1607 lth= 2 |  | 2-118 |
| 1148 - 1151 lth= 4 |  | 1-119 |  | 1645 - 1647 lth= 3 |  | 2-119 |
| 1153 - 1167 lth= 15 |  | 1-120 |  | 1651 - 1654 lth= 4 |  | 2-120 |
| 1172 - 1185 lth= 14 | **8** | 1-121 |  | 1677 - 1684 lth= 8 |  | 2-121 |
| 1186 - 1188 lth= 3 |  | 1-122 |  | 1685 - 1686 lth= 2 |  | 2-122 |
| 1191 - 1203 lth= 13 | **16** | 1-123 |  | 1689 - 1690 lth= 2 |  | 2-123 |
| 1205 - 1206 lth= 2 |  | 1-124 |  | 1698 - 1699 lth= 2 |  | 2-124 |
| 1207 - 1208 lth= 2 |  | 1-125 |  | 1701 - 1703 lth= 3 |  | 2-125 |
| 1211 - 1223 lth= 13 | **30** | 1-126 |  | 1704 - 1705 lth= 2 |  | 2-126 |
| 1227 - 1235 lth= 9 | **6** | 1-127 |  | 1706 - 1707 lth= 2 |  | 2-127 |
| 1236 - 1242 lth= 7 |  | 1-128 |  | 1708 - 1711 lth= 4 |  | 2-128 |
| 1244 - 1258 lth= 15 | **31** | 1-129 |  | 1728 - 1734 lth= 7 |  | 2-129 |
| 1274 - 1284 lth= 11 | **32** | 1-130 |  | 1737 - 1738 lth= 2 |  | 2-130 |
| 1285 - 1293 lth= 9 |  | 1-131 |  | 1740 - 1741 lth= 2 |  | 2-131 |
| 1294 - 1295 lth= 2 |  | 1-132 |  | 1748 - 1749 lth= 2 |  | 2-132 |
| 1297 - 1303 lth= 7 |  | 1-133 |  | 1751 - 1753 lth= 3 |  | 2-133 |
| 1313 - 1317 lth= 5 |  | 1-134 |  | 1754 - 1756 lth= 3 |  | 2-134 |
| 1318 - 1322 lth= 5 |  | 1-135 |  | 1772 - 1778 lth= 7 |  | 2-135 |
| 1323 - 1330 lth= 8 | **10** | 1-136 |  | 1788 - 1790 lth= 3 |  | 2-136 |
| 1331 - 1332 lth= 2 |  | 1-137 |  | 1802 - 1809 lth= 8 |  | 2-137 |
| 1344 - 1349 lth= 6 | **11** | 1-138 |  | 1811 - 1818 lth= 8 |  | 2-138 |
| 1350 - 1353 lth= 4 |  | 1-139 |  | 1823 - 1824 lth= 2 |  | 2-139 |
| 1354 - 1358 lth= 5 |  | 1-140 |  | 1853 - 1857 lth= 5 |  | 2-140 |
| 1363 - 1366 lth= 4 |  | 1-141 |  | 1858 - 1872 lth= 15 |  | 2-141 |
| 1367 - 1369 lth= 3 |  | 1-142 |  | 1873 - 1875 lth= 3 |  | 2-142 |
| 1371 - 1377 lth= 7 |  | 1-143 |  | 1879 - 1880 lth= 2 |  | 2-143 |
| 1387 - 1408 lth= 22 | **29** | 1-144 |  | 1881 - 1884 lth= 4 |  | 2-144 |
| 1411 - 1419 lth= 9 | **9** | 1-145 |  | 1885 - 1901 lth= 17 |  | 2-145 |
| 1421 - 1432 lth= 12 |  | 1-146 |  | 1902 - 1905 lth= 4 |  | 2-146 |
| 1435 - 1440 lth= 6 |  | 1-147 |  | 1906 - 1907 lth= 2 |  | 2-147 |
| 1474 - 1486 lth= 13 |  | 1-148 |  | 1909 - 1912 lth= 4 |  | 2-148 |
| 1487 - 1488 lth= 2 |  | 1-149 |  | 1916 - 1917 lth= 2 |  | 2-149 |
| 1489 - 1493 lth= 5 | **33** | 1-150 |  | 1919 - 1926 lth= 8 |  | 2-150 |
| 1494 - 1497 lth= 4 |  | 1-151 |  | 1927 - 1929 lth= 3 |  | 2-151 |
| 1498 - 1500 lth= 3 |  | 1-152 |  | 1945 - 1950 lth= 6 |  | 2-152 |
| 1510 - 1519 lth= 10 |  | 1-153 |  | 1951 - 1952 lth= 2 |  | 2-153 |
| 1521 - 1534 lth= 14 | **34** | 1-154 |  | 1955 - 1963 lth= 9 |  | 2-154 |
| 1536 - 1546 lth= 11 | **27** | 1-155 |  | 1964 - 1998 lth= 35 |  | 2-155 |
| 1547 - 1558 lth= 12 |  | 1-156 |  | 1999 - 2001 lth= 3 |  | 2-156 |
| 1569 - 1570 lth= 2 |  | 1-157 |  | 2002 - 2008 lth= 7 |  | 2-157 |
| 1581 - 1587 lth= 7 |  | 1-158 |  | 2012 - 2013 lth= 2 |  | 2-158 |
| 1594 - 1596 lth= 3 |  | 1-159 |  | 2015 - 2021 lth= 7 |  | 2-159 |
| 1603 - 1604 lth= 2 |  | 1-160 |  | 2024 - 2026 lth= 3 |  | 2-160 |
| 1618 - 1620 lth= 3 |  | 1-161 |  | 2028 - 2034 lth= 7 |  | 2-161 |
| 1678 - 1679 lth= 2 |  | 1-162 |  | 2036 - 2038 lth= 3 |  | 2-162 |
| 1690 - 1692 lth= 3 |  | 1-163 |  | 2039 - 2053 lth= 15 |  | 2-163 |
| 1697 - 1699 lth= 3 |  | 1-164 |  | 2056 - 2059 lth= 4 |  | 2-164 |
| 1701 - 1702 lth= 2 |  | 1-165 |  | 2061 - 2068 lth= 8 |  | 2-165 |
| 1703 - 1705 lth= 3 |  | 1-166 |  | 2069 - 2070 lth= 2 |  | 2-166 |
| 1706 - 1707 lth= 2 |  | 1-167 |  | 2071 - 2086 lth= 16 |  | 2-167 |
| 1711 - 1716 lth= 6 |  | 1-168 |  | 2087 - 2097 lth= 11 |  | 2-168 |
| 1720 - 1721 lth= 2 |  | 1-169 |  | 2099 - 2106 lth= 8 |  | 2-169 |
| 1735 - 1736 lth= 2 |  | 1-170 |  | 2114 - 2117 lth= 4 |  | 2-170 |
| 1737 - 1747 lth= 11 |  | 1-171 |  | 2118 - 2120 lth= 3 |  | 2-171 |
| 1748 - 1762 lth= 15 |  | 1-172 |  | 2121 - 2124 lth= 4 |  | 2-172 |
| 1763 - 1764 lth= 2 |  | 1-173 |  | 2125 - 2127 lth= 3 |  | 2-173 |
| 1770 - 1776 lth= 7 |  | 1-174 |  | 2131 - 2132 lth= 2 |  | 2-174 |
| 1777 - 1781 lth= 5 |  | 1-175 |  | 2135 - 2136 lth= 2 |  | 2-175 |
| 1783 - 1786 lth= 4 |  | 1-176 |  | 2137 - 2139 lth= 3 |  | 2-176 |
| 1790 - 1793 lth= 4 |  | 1-177 |  | 2142 - 2146 lth= 5 |  | 2-177 |
| 1794 - 1797 lth= 4 |  | 1-178 |  | 2150 - 2151 lth= 2 |  | 2-178 |
| 1801 - 1804 lth= 4 |  | 1-179 |  | 2152 - 2153 lth= 2 |  | 2-179 |
| 1805 - 1806 lth= 2 |  | 1-180 |  | 2167 - 2175 lth= 9 |  | 2-180 |
| 1807 - 1808 lth= 2 |  | 1-181 |  | 2177 - 2179 lth= 3 |  | 2-181 |
| 1809 - 1812 lth= 4 |  | 1-182 |  | 2183 - 2184 lth= 2 |  | 2-182 |
| 1813 - 1818 lth= 6 |  | 1-183 |  | 2193 - 2200 lth= 8 |  | 2-183 |
| 1820 - 1830 lth= 11 |  | 1-184 |  | 2201 - 2203 lth= 3 |  | 2-184 |
| 1831 - 1840 lth= 10 |  | 1-185 |  | 2204 - 2207 lth= 4 |  | 2-185 |
| 1841 - 1842 lth= 2 |  | 1-186 |  | 2209 - 2210 lth= 2 |  | 2-186 |
| 1843 - 1847 lth= 5 |  | 1-187 |  | 2211 - 2216 lth= 6 |  | 2-187 |
| 1848 - 1849 lth= 2 |  | 1-188 |  | 2217 - 2220 lth= 4 |  | 2-188 |
| 1850 - 1854 lth= 5 |  | 1-189 |  | 2223 - 2227 lth= 5 |  | 2-189 |
| 1855 - 2394 lth= 540 |  | 1-190 |  | 2228 - 2229 lth= 2 |  | 2-190 |
| 2396 - 2400 lth= 5 |  | 1-191 |  | 2243 - 2244 lth= 2 |  | 2-191 |
| 2401 - 2582 lth= 182 |  | 1-192 |  | 2251 - 2252 lth= 2 |  | 2-192 |
| 2583 - 2586 lth= 4 |  | 1-193 |  | 2254 - 2255 lth= 2 |  | 2-193 |
| 2587 - 2600 lth= 14 |  | 1-194 |  | 2256 - 2257 lth= 2 |  | 2-194 |
| 2601 - 2602 lth= 2 |  | 1-195 |  | 2258 - 2262 lth= 5 |  | 2-195 |
| 2603 - 2605 lth= 3 |  | 1-196 |  | 2264 - 2267 lth= 4 |  | 2-196 |
| 2606 - 2610 lth= 5 |  | 1-197 |  | 2269 - 2272 lth= 4 |  | 2-197 |
| 2611 - 2625 lth= 15 |  | 1-198 |  | 2311 - 2313 lth= 3 |  | 2-198 |
| 2627 - 2630 lth= 4 |  | 1-199 |  | 2321 - 2322 lth= 2 |  | 2-199 |
| 2631 - 2636 lth= 6 |  | 1-200 |  | 2326 - 2328 lth= 3 |  | 2-200 |
| 2637 - 2638 lth= 2 |  | 1-201 |  | 2379 - 2380 lth= 2 |  | 2-201 |
| 2658 - 2660 lth= 3 |  | 1-202 |  | 2388 - 2389 lth= 2 |  | 2-202 |
| 2662 - 2663 lth= 2 |  | 1-203 |  | 2391 - 2392 lth= 2 |  | 2-203 |
| 2677 - 2683 lth= 7 |  | 1-204 |  | 2393 - 2394 lth= 2 |  | 2-204 |
| 2684 - 2686 lth= 3 |  | 1-205 |  | 2400 - 2401 lth= 2 |  | 2-205 |
|  |  |  |  | 2402 - 2404 lth= 3 |  | 2-206 |
|  |  |  |  | 2407 - 2410 lth= 4 |  | 2-207 |
|  |  |  |  | 2411 - 2412 lth= 2 |  | 2-208 |
|  |  |  |  | 2413 - 2417 lth= 5 |  | 2-209 |
|  |  |  |  | 2425 - 2426 lth= 2 |  | 2-210 |
|  |  |  |  | 2431 - 2436 lth= 6 |  | 2-211 |
|  |  |  |  | 2437 - 2439 lth= 3 |  | 2-212 |
|  |  |  |  | 2440 - 2442 lth= 3 |  | 2-213 |
|  |  |  |  | 2443 - 2450 lth= 8 |  | 2-214 |
|  |  |  |  | 2451 - 2460 lth= 10 |  | 2-215 |
|  |  |  |  | 2461 - 2463 lth= 3 |  | 2-216 |
|  |  |  |  | 2466 - 2467 lth= 2 |  | 2-217 |
|  |  |  |  | 2478 - 2479 lth= 2 |  | 2-218 |
|  |  |  |  | 2480 - 2481 lth= 2 |  | 2-219 |
|  |  |  |  | 2486 - 2487 lth= 2 |  | 2-220 |
|  |  |  |  | 2494 - 2495 lth= 2 |  | 2-221 |
|  |  |  |  | 2507 - 2508 lth= 2 |  | 2-222 |
|  |  |  |  | 2512 - 2514 lth= 3 |  | 2-223 |
|  |  |  |  | 2515 - 2517 lth= 3 |  | 2-224 |
|  |  |  |  | 2522 - 2523 lth= 2 |  | 2-225 |
|  |  |  |  | 2531 - 2532 lth= 2 |  | 2-226 |
|  |  |  |  | 2566 - 2568 lth= 3 |  | 2-227 |
|  |  |  |  | 2577 - 2578 lth= 2 |  | 2-228 |
|  |  |  |  | 2580 - 2582 lth= 3 |  | 2-229 |
|  |  |  |  | 2670 - 2672 lth= 3 |  | 2-230 |
|  |  |  |  | 2674 - 2675 lth= 2 |  | 2-231 |
|  |  |  |  | 2681 - 2682 lth= 2 |  | 2-232 |

^a^Scan information provide first and last scan, and length of each RMS as “first – last lth =”.

**Table S2.** List of 466 plants used for the construction of an in-house database

| No | scientific name | parts | family | origin | collection  source | The number of RMSs |
| --- | --- | --- | --- | --- | --- | --- |
| **1** | *Rubus takesimensis* | root | Rosaceae | Korea | KRIBB^a^ | 212 |
| **2** | *Dystaenia takeshimana* | root | Umbelliferae | Korea | KRIBB | 133 |
| **3** | *Angelica japonica* | stem; root | Umbelliferae | Korea | KRIBB | 144 |
| **4** | *Kirengeshoma koreana* | root | Saxifragaceae | Korea | KRIBB | 133 |
| **5** | *Astilbe chinensis var. davidii* | root | Saxifragaceae | Korea | KRIBB | 128 |
| **6** | *Adonis amurensis* | whole | Ranunculaceae | Korea | KRIBB | 152 |
| **7** | *Cytomium fortunei* | root | Aspidaceae | Korea | KRIBB | 159 |
| **8** | *Phytolacca esculenta* | root | Phytolaccaceae | Korea | KRIBB | 139 |
| **9** | *Aruncus dioicus var. kamtschaticus* | stem; root | Rosaceae | Korea | KRIBB | 179 |
| **10** | *Scrophularia kakudensis* | root | Scrophulariaceae | Korea | KRIBB | 140 |
| **11** | *Lycoris aurea* | underground | Amaryllidaceae | Korea | KRIBB | 112 |
| **12** | *Campanula glomerata var. dahurica* | root | Campanulaceae | Korea | KRIBB | 143 |
| **13** | *Hemerocallis dumortieri* | root | Liliaceae | Korea | KRIBB | 137 |
| **14** | *Caulophyllum rebustum* | root | Berberidaceae | Korea | KRIBB | 157 |
| **15** | *Cimicifuga daburica* | root | Ranunculaceae | Korea | KRIBB | 143 |
| **16** | *Rodgersia podophylla* | root | Saxifragaceae | Korea | KRIBB | 145 |
| **17** | *Anthriscus sylvestris* | root | Umbelliferae | Korea | KRIBB | 114 |
| **18** | *Caltha palustris var. membranacea* | root | Ranunculaceae | Korea | KRIBB | 168 |
| **19** | *Angelica czernevia* | root | Umbelliferae | Korea | KRIBB | 120 |
| **20** | *Angelica dahurica* | root | Umbelliferae | Korea | KRIBB | 160 |
| **21** | *Spodiopogon cotulifer* | root | Gramineae | Korea | KRIBB | 96 |
| **22** | *Mirabilis jalapa* | root | Nyctaginaceae | Korea | KRIBB | 126 |
| **23** | *Xanthium strumarium* | root | Compositae | Korea | KRIBB | 156 |
| **24** | *Phytolacca insularis* | root | Phytolaccaceae | Korea | KRIBB | 191 |
| **25** | *Iris ensata var. spontanea* | root | Iridaceae | Korea | KRIBB | 185 |
| **26** | *Patrinia scabiosaefolia* | root | Valerianaceae | Korea | KRIBB | 170 |
| **27** | *Geranium kunthii* | root | Geraniaceae | Korea | KRIBB | 140 |
| **28** | *Ranunculus borealis* | root | Ranunculaceae | Korea | KRIBB | 130 |
| **29** | *Abies koreana* | root | Pinaceae | Korea | KRIBB | 127 |
| **30** | *Acer pseudo-sibolianum* | root | Aceraceae | Korea | KRIBB | 147 |
| **31** | *Koelreuteria paniculata* | root | Sapindaceae | Korea | KRIBB | 137 |
| **32** | *Cercis chinensis* | root | Leguminosae | Korea | KRIBB | 133 |
| **33** | *Adonis amurensis* | root | Ranunculaceae | Korea | KRIBB | 144 |
| **34** | *Acer triflorum* | root | Aceraceae | Korea | KRIBB | 129 |
| **35** | *Crataegus pinnatifida* | root | Rosaceae | Korea | KRIBB | 142 |
| **36** | *Zingiber mioga* | root | Zingiberaceae | Korea | KRIBB | 113 |
| **37** | *Acer okamotoanum* | root | Aceraceae | Korea | KRIBB | 135 |
| **38** | *Arctium lappa* | root | Compositae | Korea | KRIBB | 98 |
| **39** | *Chionanthus retusa* | root | Oleaceae | Korea | KRIBB | 143 |
| **40** | *Betula platyphylla var. japonica* | root | Betulaceae | Korea | KRIBB | 149 |
| **41** | *Styrax obassia* | root | Styracaceae | Korea | KRIBB | 165 |
| **42** | *Corylopsis coreana* | root | Hamamelidaceae | Korea | KRIBB | 148 |
| **43** | *Rumex conglomeratus* | root | Polygonaceae | Korea | KRIBB | 132 |
| **44** | *Corydalis filistipes* | root | Fumariaceae | Korea | KRIBB | 120 |
| **45** | *Dicentra spectabilis* | root | Fumariaceae | Korea | KRIBB | 114 |
| **46** | *Diospyros kaki* | root | Ebenaceae | Korea | KRIBB | 98 |
| **47** | *Rhodotypos scandens* | root | Rosaceae | Korea | KRIBB | 141 |
| **48** | *Isodon excisus* | root | Labiatae | Korea | KRIBB | 122 |
| **49** | *Vitex negundo var. incisa* | root | Verbenaceae | Korea | KRIBB | 166 |
| **50** | *Lycium chinense* | root | Solanaceae | Korea | KRIBB | 136 |
| **51** | *Helianthus tuberosus* | root | Compositae | Korea | KRIBB | 161 |
| **52** | *Helianthus tuberosus* | tuber | Compositae | Korea | KRIBB | 146 |
| **53** | *Ricinus communis* | root | Euphorbiaceae | Korea | KRIBB | 201 |
| **54** | *Lathyrus davidii* | root | Leguminosae | Korea | KRIBB | 137 |
| **55** | *Boehmeria pannosa* | root | Urticaceae | Korea | KRIBB | 133 |
| **56** | *Echinops setifer* | root | Compositae | Korea | KRIBB | 149 |
| **57** | *Ginkgo biloba* | root | Ginkgoaceae | Korea | KRIBB | 128 |
| **58** | *Mirabilis jalapa* | root | Nyctaginaceae | Korea | KRIBB | 205 |
| **59** | *Clematis apiifolia* | root | Ranunculaceae | Korea | KRIBB | 207 |
| **60** | *Pinus thunbergii* | root | Pinaceae | Korea | KRIBB | 183 |
| **61** | *Fagus crenata var. multinervis* | root | Fagaceae | Korea | KRIBB | 128 |
| **62** | *Morus bombycis* | root | Moraceae | Korea | KRIBB | 117 |
| **63** | *Ulmus laciniata* | root | Ulmaceae | Korea | KRIBB | 127 |
| **64** | *Liriope platyphylla* | tuber | Liliaceae | Korea | KRIBB | 173 |
| **65** | *Tilia insularis* | root | Tiliaceae | Korea | KRIBB | 129 |
| **66** | *Pinus densiflora* | root | Pinaceae | Korea | KRIBB | 151 |
| **67** | *Citrus junos* | root | Rutaceae | Korea | KRIBB | 156 |
| **68** | *Pinus koraiensis* | root | Pinaceae | Korea | KRIBB | 131 |
| **69** | *Aralia elata* | root | Araliaceae | Korea | KRIBB | 141 |
| **70** | *Clematis heracleifolia* | root | Ranunculaceae | Korea | KRIBB | 126 |
| **71** | *Dystaenia takeshimana* | root | Umbelliferae | Korea | KRIBB | 184 |
| **72** | *Actinidia polygama* | stem; root | Actinidiaceae | Korea | KRIBB | 161 |
| **73** | *Phytolacca americana* | root | Phytolaccaceae | Korea | KRIBB | 158 |
| **74** | *Panax ginseng* | root | Araliaceae | Korea | KRIBB | 109 |
| **75** | *Panax ginseng* | root | Araliaceae | Korea | KRIBB | 167 |
| **76** | *Disporum sessile* | root | Liliaceae | Korea | KRIBB | 166 |
| **77** | *Capsicum baccatum* | root | Solanaceae | Korea | KRIBB | 116 |
| **78** | *Capsicum chinense* | root | Solanaceae | Korea | KRIBB | 130 |
| **79** | *Barbarea orthoceras* | root | Cruciferae | Korea | KRIBB | 133 |
| **80** | *Cyrtomium falcatum* | root | Aspidaceae | Korea | KRIBB | 116 |
| **81** | *Aralia continentalis* | root | Araliaceae | Korea | KRIBB | 135 |
| **82** | *Angelica dahurica* | root | Umbelliferae | Korea | KRIBB | 219 |
| **83** | *Fortunella japonica var. margarita* | whole | Rutaceae | Korea | KRIBB | 131 |
| **84** | *Helianthus tuberosus* | root | Compositae | Korea | KRIBB | 133 |
| **85** | *Leonurus sibiricus* | root | Labiatae | Korea | KRIBB | 140 |
| **86** | *Ficus elastica* | whole | Moraceae | Korea | KRIBB | 130 |
| **87** | *Plantago asiatica* | root | Plantaginaceae | Korea | KRIBB | 123 |
| **88** | *Rubus coreanus* | root | Rosaceae | Korea | KRIBB | 155 |
| **89** | *Rhus chinensis* | root | Anacardiaceae | Korea | KRIBB | 162 |
| **90** | *Rosa multiflora* | root | Rosaceae | Korea | KRIBB | 168 |
| **91** | *Sorbus commixta* | root | Rosaceae | Korea | KRIBB | 157 |
| **92** | *Persicaria nodosa* | whole | Polygonaceae | Korea | KRIBB | 151 |
| **93** | *Gladiolus gandavensis Van* | root | Iridaceae | Korea | KRIBB | 151 |
| **94** | *Nelumbo nucifera* | root | Nymphaeaceae | Korea | KRIBB | 153 |
| **95** | *Dryopteris crassirhizoma* | root | Aspidaceae | Korea | KRIBB | 153 |
| **96** | *Aster ageratoides* | root | Compositae | Korea | KRIBB | 197 |
| **97** | *Agastache rugosa* | root | Labiatae | Korea | KRIBB | 154 |
| **98** | *Sonchus brachyotus* | root | Compositae | Korea | KRIBB | 139 |
| **99** | *Filipendula formosa* | root | Rosaceae | Korea | KRIBB | 167 |
| **100** | *Lysimachia clethroides* | root | Primulaceae | Korea | KRIBB | 134 |
| **101** | *Pueraria lobata* | flower | Leguminosae | China | KRIBB | 145 |
| **102** | *Chrysanthemum indicum* | flower | Compositae | China | KRIBB | 118 |
| **103** | *Ostericum koreanum* | root | Umbelliferae | Korea | KRIBB | 179 |
| **104** | *Liriope muscari* | tuber | Liliaceae | China | KRIBB | 133 |
| **105** | *Polygala tenuifolia* | root | Polygalaceae | China | KRIBB | 132 |
| **106** | *Castanea crenata* | seed | Fagaceae | Korea | KRIBB | 168 |
| **107** | *Rehmannia glutinosa* | root | Scrophulariaceae | Korea | KRIBB | 144 |
| **108** | *Drynaria fortunei* | root | Davalliaceae | China | KRIBB | 93 |
| **109** | *Pogostemon cablin* | whole | Labiatae | China | KRIBB | 150 |
| **110** | *Sophora japonica* | fruits | Leguminosae | China | KRIBB | 136 |
| **111** | *Dianthus chinensis* | whole | Caryophyllaceae | China | KRIBB | 105 |
| **112** | *Euonymus alatus* | twig | Celastraceae | China | KRIBB | 123 |
| **113** | *Citrus unshiu* | pericarp | Rutaceae | Korea | KRIBB | 120 |
| **114** | *Citrus reticulata* | seed | Rutaceae | China | KRIBB | 105 |
| **115** | *Cibotium barometz* | root | Dicksoniaceae | China | KRIBB | 136 |
| **116** | *Lysimachia christinae* | whole | Leguminosae | China | KRIBB | 137 |
| **117** | *Playtcodon grandiflorum* | root | Campanulaceae | China | KRIBB | 145 |
| **118** | *Trachelospermum asiaticum* | stem | Apocynaceae | China | KRIBB | 124 |
| **119** | *Phragmites communis* | root | Gramineae | China | KRIBB | 142 |
| **120** | *Aloe ferox, Aloe africana, Aloe spicata* | whole | Liliaceae | China | KRIBB | 157 |
| **121** | *Angelica sinensis* | root | Umbelliferae | China | KRIBB | 136 |
| **122** | *Angelica gigas* | main root | Umbelliferae | Korea | KRIBB | 135 |
| **123** | *Angelica gigas* | root | Umbelliferae | Korea | KRIBB | 144 |
| **124** | *Angelica gigas* | rootlet | Umbelliferae | Korea | KRIBB | 146 |
| **125** | *Aconitum carmichaeli* | tuber | Ranunculaceae | China | KRIBB | 131 |
| **126** | *Areca catechu* | pericarp | Palmae | China | KRIBB | 99 |
| **127** | *Prunus persica* | seed | Rosaceae | China | KRIBB | 104 |
| **128** | *Prunus persica* | seed | Rosaceae | China | KRIBB | 147 |
| **129** | *Eucommia ulmoides* | bark | Eucommiaceae | Korea | KRIBB | 85 |
| **130** | *Eucommia ulmoides* | leave | Eucommiaceae | Korea | KRIBB | 115 |
| **131** | *Eucommia ulmoides* | twig | Eucommiaceae | Korea | KRIBB | 104 |
| **132** | *Eucommia ulmoides* | bark | Eucommiaceae | Korea | KRIBB | 117 |
| **133** | *Polygonatum odoratum* | root | Asparagaceae | Korea | KRIBB | 151 |
| **134** | *Aristolochia debilis* | fruits | Aristolochiaceae | China | KRIBB | 127 |
| **135** | *Cannabis sativa* | seed | Cannabinaceae | China | KRIBB | 127 |
| **136** | *Strychnos nux-vomica* | seed | Loganiaceae | China | KRIBB | 141 |
| **137** | *Ephedra* | whole | Ephedraceae | China | KRIBB | 113 |
| **138** | *Codonopsis pilosula* | root | Campanulaceae | China | KRIBB | 118 |
| **139** | *Vitex rotundifolia* | fruits | Verbenaceae | China | KRIBB | 125 |
| **140** | *Liriope muscari* | tuber | Liliaceae | China | KRIBB | 148 |
| **141** | *Pseudocydonia sinensis* | fruits | Rosaceae | Korea | KRIBB | 165 |
| **142** | *Fraxinus rhynchophylla* | bark | Oleaceae | China | KRIBB | 159 |
| **143** | *Saussurea costus* | root | Compositae | China | KRIBB | 127 |
| **144** | *Actinidia chinensis* | fruits | Actinidiaceae | China | KRIBB | 122 |
| **145** | *Actinidia chinensis* | stem | Actinidiaceae | China | KRIBB | 119 |
| **146** | *Buddleja officinalis* | flower | Loganiaceae | China | KRIBB | 162 |
| **147** | *Mentha canadensis* | whole | Labiatae | China | KRIBB | 126 |
| **148** | *Sterculia lychnophora* | seed | Sterculiaceae | China | KRIBB | 149 |
| **149** | *Scutellaria barbata* | whole | Labiatae | China | KRIBB | 130 |
| **150** | *Pulsatilla koreana* | root | Ranunculaceae | China | KRIBB | 160 |
| **151** | *Cynanchum atratum* | root | Asclepiadaceae | China | KRIBB | 139 |
| **152** | *Stemona japonica* | root | Stemonaceae | China | KRIBB | 110 |
| **153** | *Ampelopsis japonica* | root | Vitaceae | China | KRIBB | 147 |
| **154** | *Paeonia japonica* | root | Ranunculaceae | Korea | KRIBB | 169 |
| **155** | *Tribulus terrestris* | seed | Zygophyllaceae | China | KRIBB | 159 |
| **156** | *Tribulus terrestris* | seed | Zygophyllaceae | China | KRIBB | 87 |
| **157** | *Pharbitis nil* | seed | Compositae | China | KRIBB | 155 |
| **158** | *Atractylodes japonica* | root | Compositae | China | KRIBB | 135 |
| **159** | *Atractylodes japonica* | root | Compositae | China | KRIBB | 118 |
| **160** | *Dolichos lablab* | seed | Leguminosae | China | KRIBB | 121 |
| **161** | *Dolichos lablab* | seed | Leguminosae | China | KRIBB | 140 |
| **162** | *Pleuropterus multiflorus* | root | Asclepiadaceae | China | KRIBB | 173 |
| **163** | *Pleuropterus multiflorus* | root | Asclepiadaceae | Korea | KRIBB | 179 |
| **164** | *Lilium lancifolium* | bulb | Liliaceae | China | KRIBB | 150 |
| **165** | *Rubus coreanus* | fruits | Rosaceae | China | KRIBB | 200 |
| **166** | *Rubus coreanus* | fruits | Rosaceae | Korea | KRIBB | 174 |
| **167** | *Curcuma zedoaria* | root | Zingiberaceae | China | KRIBB | 95 |
| **168** | *Triticum aestivum* | seed | Gramineae | China | KRIBB | 128 |
| **169** | *Spirodela polyrrhiza* | whole | Lemnaceae | China | KRIBB | 125 |
| **170** | *Dioscorea tokora* | root | Dioscoreaceae | China | KRIBB | 110 |
| **171** | *Areca catechu* | seed | Palmae | China | KRIBB | 81 |
| **172** | *Belamcanda chinensis* | root | Iridaceae | China | KRIBB | 181 |
| **173** | *Luffa cylindrica* | fruits | Cucurbitaceae | China | KRIBB | 176 |
| **174** | *Combretum indicum* | fruits | Combretaceae | China | KRIBB | 162 |
| **175** | *Codonopsis lanceolata* | root | Campanulaceae | China | KRIBB | 143 |
| **176** | *Adenophora triphylla* | root | Campanulaceae | Korea | KRIBB | 121 |
| **177** | *Torilis japonica* | fruits | Umbelliferae | Korea | KRIBB | 49 |
| **178** | *Crataegus pinnatifida* | fruits | Rosaceae | China | KRIBB | 221 |
| **179** | *Pueraria lobata* | root | Leguminosae | Korea | KRIBB | 128 |
| **180** | *Nardostachys chinensis* | root | Valerianaceae | China | KRIBB | 125 |
| **181** | *Euphorbia kansui* | root | Euphorbiaceae | China | KRIBB | 182 |
| **182** | *Dalbergia odorifera* | stem, root | Leguminosae | China | KRIBB | 72 |
| **183** | *Curcuma longa* | root | Zingiberaceae | Korea | KRIBB | 75 |
| **184** | *Zingiber officinale* | root | Zingiberaceae | Korea | KRIBB | 59 |
| **185** | *Rehmannia glutinosa* | root | Scrophulariaceae | China | KRIBB | 141 |
| **186** | *Cassia obtusifolia* | seed | Leguminosae | China | KRIBB | 184 |
| **187** | *Cinnamomum verum* | bark | Lauraceae | China | KRIBB | 120 |
| **188** | *Alpinia officinarum* | root | Zingiberaceae | China | KRIBB | 106 |
| **189** | *Ligusticum sinensis* | root | Umbelliferae | China | KRIBB | 124 |
| **190** | *Angelica tenuissim* | root | Umbelliferae | Korea | KRIBB | 155 |
| **191** | *Sophora flavescens* | root | Leguminosae | China | KRIBB | 122 |
| **192** | *Tussilago farfara* | flower | Compositae | China | KRIBB | 137 |
| **193** | *Dryopteris crassirhizoma* | root | Aspidiaceae | China | KRIBB | 174 |
| **194** | *Trichosanthes kirilowii* | seed | Cucurbitaceae | China | KRIBB | 112 |
| **195** | *Lycium chinense* | fruits | Solanaceae | Korea | KRIBB | 123 |
| **196** | *Allium tuberosum* | seed | Liliaceae | China | KRIBB | 117 |
| **197** | *Selaginella tamariscina* | whole | Selaginellaceae | Korea | KRIBB | 156 |
| **198** | *Rosa laevigata* | fruits | Rosaceae | China | KRIBB | 187 |
| **199** | *Lonicera japonica* | flower | Caprifoliaceae | China | KRIBB | 146 |
| **200** | *Rhaponticum uniflorum* | root | Compositae | China | KRIBB | 164 |
| **201** | *Cirsium japonicum* | root | Compositae | Korea | KRIBB | 127 |
| **202** | *Portulaca oleracea* | whole | Portulacaceae | China | KRIBB | 89 |
| **203** | *Gossypium indicum* | seed | Malvaceae | China | KRIBB | 122 |
| **204** | *Momordica cochinchinensis* | seed | Cucurbitaceae | China | KRIBB | 103 |
| **205** | *Dictamnus dasycarpus* | root | Rutaceae | China | KRIBB | 154 |
| **206** | *Paeonia japonica* | root | Ranunculaceae | Korea | KRIBB | 205 |
| **207** | *Strychnos ignatii* | seed | Loganiaceae | China | KRIBB | 132 |
| **208** | *Aconitum carmichaeli* | tuber | Ranunculaceae | China | KRIBB | 68 |
| **209** | *Eriobotrya japonica* | leave | Rosaceae | China | KRIBB | 173 |
| **210** | *Amomum villosum* | seed | Zingiberaceae | China | KRIBB | 104 |
| **211** | *Crataegus pinnatifida* | fruits | Rosaceae | China | KRIBB | 178 |
| **212** | *Crataegus pinnatifida* | fruits | Rosaceae | Korea | KRIBB | 146 |
| **213** | *Cornus officinalis* | fruits | Cornaceae | China | KRIBB | 152 |
| **214** | *Cornus officinalis* | fruits | Cornaceae | Korea | KRIBB | 131 |
| **215** | *Zizyphus jujuba* | seed | Rhamnaceae | China | KRIBB | 124 |
| **216** | *Zizyphus jujuba* | seed | Rhamnaceae | China | KRIBB | 132 |
| **217** | *Sparganium stoloniferum* | root | Sparganiaceae | China | KRIBB | 131 |
| **218** | *Taxillus chinensis* | twig | Loranthaceae | China | KRIBB | 115 |
| **219** | *Morus alba* | bark | Moraceae | China | KRIBB | 131 |
| **220** | *Phytolacca acinosa* | root | Phytolaccae | China | KRIBB | 141 |
| **221** | *Dendrobium moniliforme* | whole | Orchidaceae | China | KRIBB | 106 |
| **222** | *Pyrrosia lingua* | leave | Polypodiaceae | China | KRIBB | 124 |
| **223** | *Acorus gramineus* | root | Araceae | China | KRIBB | 72 |
| **224** | *Acorus gramineus* | root | Araceae | China | KRIBB | 86 |
| **225** | *Curculigo orchioides* | root | Amaryllidaceae | China | KRIBB | 125 |
| **226** | *Inula japonica* | flower | Compositae | China | KRIBB | 162 |
| **227** | *Agrimonia pilosa* | whole | Rosaceae | China | KRIBB | 163 |
| **228** | *Triticum aestivum* | seed | Gramineae | China | KRIBB | 117 |
| **229** | *Caesalpinia sappan* | stem | Leguminosae | China | KRIBB | 114 |
| **230** | *Perilla frutescens* | leave | Labiatae | Korea | KRIBB | 135 |
| **231** | *Siebold et Zuccarini* | pollen | Pinaceae | Korea | KRIBB | 121 |
| **232** | *Ruprecht* | whole | Cynomoriaceae | China | KRIBB | 145 |
| **233** | *Cimicifuga heracleifolia* | root | Ranunculaceae | China | KRIBB | 160 |
| **234** | *Anethum graveolens* | fruits | Umbelliferae | China | KRIBB | 151 |
| **235** | *Bupleurum falcatum* | root | Umbelliferae | China | KRIBB | 146 |
| **236** | *Magnolia denudata* | flower | Magnoliaceae | China | KRIBB | 71 |
| **237** | *Houttuynia cordata* | whole | Saururaceae | China | KRIBB | 102 |
| **238** | *Veratrum nigrum* | root | Liliaceae | China | KRIBB | 100 |
| **239** | *Ligustrum lucidum* | fruits | Oleaceae | China | KRIBB | 138 |
| **240** | *Forsythia suspensa* | fruits | Oleaceae | China | KRIBB | 126 |
| **241** | *Forsythia suspensa* | fruits | Oleaceae | Korea | KRIBB | 121 |
| **242** | *Nelumbo nucifera* | seed | Nymphaceae | China | KRIBB | 186 |
| **243** | *Lysimachia foenum-graeci* | whole | Primulaceae | China | KRIBB | 124 |
| **244** | *Rosa multiflora* | fruits | Rosaceae | China | KRIBB | 147 |
| **245** | *Acanthopanax sessiliflorum* | bark | Araliaceae | China | KRIBB | 140 |
| **246** | *Prunus mume* | fruits | Rosaceae | China | KRIBB | 159 |
| **247** | *Schisandra chinensis* | fruits | Magnoliaceae | China | KRIBB | 161 |
| **248** | *Evodia rutaecarpa* | fruits | Rutaceae | China | KRIBB | 136 |
| **249** | *Prunus humillis* | seed | Rosaceae | Korea | KRIBB | 140 |
| **250** | *Polygala tenuifolia* | root | Polygalaceae | China | KRIBB | 160 |
| **251** | *Polygala tenuifolia* | root | Polygalaceae | China | KRIBB | 179 |
| **252** | *Epimedium koreanum* | whole | Berberidaceae | Korea | KRIBB | 165 |
| **253** | *Syzygium aromaticum* | flower | Myrtaceae | China | KRIBB | 117 |
| **254** | *Poncirus trifoliata* | fruits | Rutaceae | China | KRIBB | 159 |
| **255** | *Rehmannia glutinosa* | root | Scrophulariaceae | China | KRIBB | 104 |
| **256** | *Rehmannia glutinosa* | root | Scrophulariaceae | China | KRIBB | 116 |
| **257** | *Dipsacus asperoides* | root | Dipsacaceae | China | KRIBB | 174 |
| **258** | *Dipsacus asperoides* | root | Dipsacaceae | China | KRIBB | 155 |
| **259** | *Morinda officinalis* | root | Rubiaceae | China | KRIBB | 98 |
| **260** | *Acanthopanax sessiliflorum* | bark | Araliaceae | China | KRIBB | 118 |
| **261** | *Spatholobus suberectus* | stem | Leguminosae | China | KRIBB | 116 |
| **262** | *Cucumis melo L.* | calyx | Cucurbitaceae | Korea | KRIBB | 102 |
| **263** | *Raphanus sativus* | seed | Cruciferae | China | KRIBB | 115 |
| **264** | *Hydnocarpus anthelmintica* | seed | Flacourtiaceae | China | KRIBB | 93 |
| **265** | *Liriope platyphylla* | tuber | Liliaceae | Korea | KRIBB | 124 |
| **266** | *Commiphora molmol* | resin | Burseraceae | China | KRIBB | 55 |
| **267** | *Saposhnikovia divaricata* | root | Umbelliferae | Korea | KRIBB | 120 |
| **268** | *Paeonia lactiflora* | root | Ranunculaceae | China | KRIBB | 159 |
| **269** | *Poria cocos* | bark | Polyporaceae | China | KRIBB | 153 |
| **270** | *Adenophora triphylla* | root | Campanulaceae | China | KRIBB | 150 |
| **271** | *Sophora tonkinensis* | root | Leguminosae | China | KRIBB | 137 |
| **272** | *Perilla frutescens* | seed | Labiatae | China | KRIBB | 121 |
| **273** | *Triticum aestivum* | etc | Gramineae | Korea | KRIBB | 151 |
| **274** | *Linum usitatissimum* | seed | Linaceae | China | KRIBB | 128 |
| **275** | *Lindera strichnifolia* | root | Lauraceae | China | KRIBB | 117 |
| **276** | *Melandryum firmum* | whole | Caryophyllaceae | China | KRIBB | 121 |
| **277** | *Dryobalanops aromatica* | resin | Dipterocarpaceae | China | KRIBB | 51 |
| **278** | *Prunus japonica* | seed | Rosaceae | China | KRIBB | 178 |
| **279** | *Curcuma longa* | root | Zingiberaceae | China | KRIBB | 84 |
| **280** | *Potentilla chinensis* | root | Rosaceae | China | KRIBB | 176 |
| **281** | *Cinnamomum cassia* | bark | Lauraceae | China | KRIBB | 105 |
| **282** | *Epimedium brevicornum* | whole | Berberidaceae | China | KRIBB | 165 |
| **283** | *Alpinia oxyphylla* | seed | Zingiberaceae | China | KRIBB | 45 |
| **284** | *Panax ginseng* | root | Araliaceae | Korea | KRIBB | 130 |
| **285** | *Aster tataricus* | root | Compositae | China | KRIBB | 128 |
| **286** | *Lithospermum erythrorhizon* | root | Boraginaceae | China | KRIBB | 99 |
| **287** | *Ailanthus altissima* | bark | Simaroubaceae | China | KRIBB | 116 |
| **288** | *Broussonetia papyrifera* | fruits | Moraceae | China | KRIBB | 100 |
| **289** | *Paeonia lactiflora* | root | Ranunculaceae | Korea | KRIBB | 147 |
| **290** | *Peucedanum praeruptorum* | root | Umbelliferae | China | KRIBB | 124 |
| **291** | *Angelica decursiva* | root | Umbelliferae | Korea | KRIBB | 111 |
| **292** | *Lepidium apetalum* | seed | Cruciferae | China | KRIBB | 137 |
| **293** | *Syringa patula* | bark | Myrtaceae | China | KRIBB | 94 |
| **294** | *Gleditsia sinensis* | fruits | Leguminosae | China | KRIBB | 154 |
| **295** | *Lophatherum gracile* | leave | Gramineae | China | KRIBB | 109 |
| **296** | *Citrus aurantiu* | fruits | Rutaceae | China | KRIBB | 137 |
| **297** | *Anemarrhena asphodeloides* | root | Liliaceae | China | KRIBB | 160 |
| **298** | *Anemarrhena asphodeloides* | root | Liliaceae | China | KRIBB | 148 |
| **299** | *Kochia scoparia* | fruits | Chenopodiaceae | China | KRIBB | 132 |
| **300** | *Gentiana macrophylla* | root | Gentianaceae | China | KRIBB | 142 |
| **301** | *Atractylodes lancea* | root | Compositae | China | KRIBB | 130 |
| **302** | *Cnidium officinale* | root | Umbelliferae | Korea | KRIBB | 139 |
| **303** | *Aconitum carmichaeli* | tuber | Ranunculaceae | China | KRIBB | 172 |
| **304** | *Alpinia katsumadai* | seed | Zingiberaceae | China | KRIBB | 118 |
| **305** | *Gardenia jasminoides* | fruits | Rubiaceae | Korea | KRIBB | 165 |
| **306** | *Illicium verum* | fruits | Illiciaceae | China | KRIBB | 96 |
| **307** | *Albizzia julibrissin* | bark | Leguminosae | China | KRIBB | 115 |
| **308** | *Lygodium japonicum* | pollen | Schizaeaceae | China | KRIBB | 144 |
| **309** | *Kalopanax pictus* | bark | Araliaceae | China | KRIBB | 110 |
| **310** | *Prunus armeniaca* | seed | Rosaceae | China | KRIBB | 145 |
| **311** | *Cyperus rotundus* | root | Cyperaceae | China | KRIBB | 128 |
| **312** | *Cyperus rotundus* | root | Cyperaceae | Korea | KRIBB | 132 |
| **313** | *Cyperus rotundus* | root | Cyperaceae | Korea | KRIBB | 133 |
| **314** | *Elsholtzia ciliata* | whole | Labiatae | China | KRIBB | 108 |
| **315** | *Scrophularia buergeriana* | root | Scrophulariaceae | China | KRIBB | 142 |
| **316** | *Scrophularia buergeriana* | root | Scrophulariaceae | China | KRIBB | 153 |
| **317** | *Schizonepeta tenuifolia* | whole | Labiatae | China | KRIBB | 116 |
| **318** | *Schizonepeta tenuifolia* | whole | Labiatae | Korea | KRIBB | 107 |
| **319** | *Trigonella foenum-graecum* | seed | Leguminosae | China | KRIBB | 146 |
| **320** | *Polygonum cuspidatum* | root | Polygonaceae | China | KRIBB | 133 |
| **321** | *Prunus tomentosa* | bark | Rosaceae | China | KRIBB | 116 |
| **322** | *Zingiber officinale* | root | Zingiberaceae | China | KRIBB | 117 |
| **323** | *Lycium barbarum* | fruits | Solanaceae | China | KRIBB | 118 |
| **324** | *Platycodon grandiflorum* | root | Campanulaceae | Korea | KRIBB | 138 |
| **325** | *Platycodon grandiflorum* | seed | Cruciferae | China | KRIBB | 119 |
| **326** | *Platycodon grandiflorum* | seed | Cruciferae | China | KRIBB | 138 |
| **327** | *Rheum palmatum* | root | Polygonaceae | China | KRIBB | 169 |
| **328** | *Rheum officinale* | root | Polygonaceae | China | KRIBB | 154 |
| **329** | *Benincasa cerifera* | seed | Cucurbitaceae | Korea | KRIBB | 132 |
| **330** | *Malva verticillata* | seed | Malvaceae | China | KRIBB | 152 |
| **331** | *Hordeum vulgare* | fruits | Gramineae | China | KRIBB | 113 |
| **332** | *Pinellia ternata* | tuber | Araceae | China | KRIBB | 147 |
| **333** | *Thuja orientalis* | seed | Cupressaceae | China | KRIBB | 50 |
| **334** | *Hedyotis diffusa* | whole | Rubiaceae | China | KRIBB | 118 |
| **335** | *Amomum villosum* | seed | Zingiberaceae | China | KRIBB | 86 |
| **336** | *Morus alba* | fruits | Moraceae | China | KRIBB | 67 |
| **337** | *Morus alba* | twig | Moraceae | Korea | KRIBB | 123 |
| **338** | *Asiasarum heterotropoides* | root | Aristolochiaceae | China | KRIBB | 112 |
| **339** | *Pinus densiflora* | twig | Pinaceae | Korea | KRIBB | 127 |
| **340** | *Schisandra chinensis* | fruits | Magnoliaceae | Korea | KRIBB | 175 |
| **341** | *Zea mays* | etc | Gramineae | China | KRIBB | 116 |
| **342** | *Dimocarpus longan* | pericarp | Sapindaceae | Vientnam | KRIBB | 98 |
| **343** | *Artemisia iwayomogi* | whole | Compositae | Korea | KRIBB | 151 |
| **344** | *Vigna angularis* | seed | Leguminosae | China | KRIBB | 155 |
| **345** | *Erycibe obtusifolia* | stem | Convolvulaceae | China | KRIBB | 158 |
| **346** | *Rehmannia glutinosa* | root | Scrophulariaceae | China | KRIBB | 170 |
| **347** | *Plantago asiatica* | seed | Plantaginaceae | China | KRIBB | 162 |
| **348** | *Gastrodia elata* | root | Orchidaceae | China | KRIBB | 173 |
| **349** | *Harpagophytum procumbens* | whole | Pedaliaceae | China | KRIBB | 129 |
| **350** | *Rubia akane* | pericarp | Rutaceae | China | KRIBB | 119 |
| **351** | *Inula helenium* | root | Compositae | China | KRIBB | 104 |
| **352** | *Smilax china* | root | Liliaceae | China | KRIBB | 152 |
| **353** | *Tetrapanax papyriferus* | stem | Lardizabalaceae | China | KRIBB | 117 |
| **354** | *Isatis indigotica* | root | Cruciferae | China | KRIBB | 146 |
| **355** | *Patrinia scabiosaefolia* | root | Valerianaceae | China | KRIBB | 177 |
| **356** | *Vitis vinifera* | whole | Compositae | China | KRIBB | 120 |
| **357** | *Vitis vinifera* | stem | Vitaceae | Korea | KRIBB | 113 |
| **358** | *Typha orientalis* | pollen | Typhaceae | China | KRIBB | 135 |
| **359** | *Chaenomeles speciosa* | root | Cucurbitaceae | China | KRIBB | 161 |
| **360** | *Geranium thunbergii* | whole | Geraniaceae | China | KRIBB | 160 |
| **361** | *Corydalis ternata* | tuber | Papaveraceae | China | KRIBB | 136 |
| **362** | *Carthamus tinctorius* | seed | Compositae | China | KRIBB | 126 |
| **363** | *Scutellaria baicalensis* | root | Scrophulariaceae | China | KRIBB | 170 |
| **364** | *Astragalus membranaceus* | root | Leguminosae | China | KRIBB | 132 |
| **365** | *Lindera obtusiloba* | twig | Lauraceae | China | KRIBB | 96 |
| **366** | *Pharbitis purpurea* | seed | Compositae | China | KRIBB | 134 |
| **367** | *Siegesbeckia pubescens* | whole | Compositae | Korea | KRIBB | 116 |
| **368** | *Pogostemon cablin* | flower | Labiatae | Korea | SNUG^b^ | 146 |
| **369** | *Pogostemon cablin* | leaves, twigs | Labiatae | Korea | SNUG | 182 |
| **370** | *Hosta japonica var. lancifolia* | flower | Liliaceae | Korea | SNUG | 166 |
| **371** | *Hosta japonica var. lancifolia* | leaves, twigs | Liliaceae | Korea | SNUG | 190 |
| **372** | *Lotus corniculatus var. japonica* | whole | Fabaceae | Korea | SNUG | 169 |
| **373** | *Artemisia annua* | whole | Compositae | Korea | SNUG | 136 |
| **374** | *Aeschynomene indica* | fruits | Fabaceae | Korea | SNUG | 121 |
| **375** | *Aeschynomene indica* | whole | Fabaceae | Korea | SNUG | 133 |
| **376** | *Cassia occidentalis* | fruits | Fabaceae | Korea | SNUG | 147 |
| **377** | *Cassia occidentalis* | leaves, twigs, flower | Fabaceae | Korea | SNUG | 142 |
| **378** | *Cassia obtusifolia* | fruits | Fabaceae | Korea | SNUG | 131 |
| **379** | *Cassia obtusifolia* | leaves, twigs, flower | Fabaceae | Korea | SNUG | 153 |
| **380** | *Celastrus orbiculatus* | leaves, stem | Celastraceae | Korea | SNUG | 138 |
| **381** | *Trifolium pratense* | flower | Fabaceae | Korea | SNUG | 140 |
| **382** | *Trifolium pratense* | leaves, stem | Fabaceae | Korea | SNUG | 146 |
| **383** | *Elsholtzia ciliata* | flower | Labiatae | Korea | SNUG | 150 |
| **384** | *Elsholtzia ciliata* | leaves, stem | Labiatae | Korea | SNUG | 153 |
| **385** | *Cacalia firma* | leaves | Compositae | Korea | SNUG | 130 |
| **386** | *Kummerowia striata* | whole | Fabaceae | Korea | SNUG | 169 |
| **387** | *Clematis mandshurica* | whole | Ranunculaceae | Korea | SNUG | 217 |
| **388** | *Thalictrum aquilegifolium* | whole | Ranunculaceae | Korea | SNUG | 138 |
| **389** | *Viburnum sargentii* | whole | Caprifoliaceae | Korea | SNUG | 169 |
| **390** | *Lagerstroemia indica* | leaves, twigs | Lythraceae | Korea | SNUG | 138 |
| **391** | *Veronicastrum sibiricum var. yezonense* | whole | Scrophulariaceae | Korea | SNUG | 210 |
| **392** | *Serratula coronata var. insularis* | whole | Compositae | Korea | SNUG | 146 |
| **393** | *Sambucus sieboldoldiana var. pendula* | leaves, twigs | Caprifoliaceae | Korea | SNUG | 173 |
| **394** | *Serratula coronata var. insularis* | flower | Compositae | Korea | SNUG | 171 |
| **395** | *Akebia quinata* | whole | Lardizabalaceae | Korea | SNUG | 210 |
| **396** | *Aruncus dioicus var. kamtschaticus* | whole | Rosaceae | Korea | SNUG | 178 |
| **397** | *Aster tataricus* | flower | Compositae | Korea | SNUG | 136 |
| **398** | *Hylotelephium ussuriense* | whole | Crassulaceae | Korea | SNUG | 146 |
| **399** | *Amorpha fruticosa* | leaves, twigs | Fabaceae | Korea | SNUG | 146 |
| **400** | *Amorpha fruticosa* | fruits | Fabaceae | Korea | SNUG | 174 |
| **401** | *Abeliophyllum distichum* | leaves, twigs | Oleaceae | Korea | SNUG | 172 |
| **402** | *Callicarpa japonica* | fruits | Verbenaceae | Korea | SNUG | 160 |
| **403** | *Callicarpa japonica* | leaves, twigs | Verbenaceae | Korea | SNUG | 170 |
| **404** | *Angelica decursiva* | whole | Umbelliferae | Korea | SNUG | 163 |
| **405** | *Spiraea microgyna* | whole | Rosaceae | Korea | SNUG | 174 |
| **406** | *Sedum middendorffianum* | whole | Crassulaceae | Korea | SNUG | 174 |
| **407** | *Cnidium officinale* | whole | Umbelliferae | Korea | SNUG | 128 |
| **408** | *Ligusticum chuanxiong* | whole | Umbelliferae | Korea | SNUG | 177 |
| **409** | *Scutellaria baicalensis* | flower, whole | Labiatae | Korea | SNUG | 141 |
| **410** | *Aster sphathulifolius* | whole | Compositae | Korea | SNUG | 122 |
| **411** | *Angelica dahurica* | fruits | Umbelliferae | Korea | SNUG | 138 |
| **412** | *Glehnia littoralis* | whole | Umbelliferae | Korea | SNUG | 143 |
| **413** | *Angelica gigas* | fruits | Umbelliferae | Korea | SNUG | 125 |
| **414** | *Angelica gigas* | leaves | Umbelliferae | Korea | SNUG | 153 |
| **415** | *Leonurus macranthus* | whole | Labiatae | Korea | SNUG | 118 |
| **416** | *Crataegus pinnatifida* | leaves | Rosaceae | Korea | SNUG | 146 |
| **417** | *Crataegus pinnatifida* | fruits | Rosaceae | Korea | SNUG | 172 |
| **418** | *Crataegus pinnatifida* | twigs | Rosaceae | Korea | SNUG | 158 |
| **419** | *Ilex serrata* | fruits | Aquifoliaceae | Korea | SNUG | 184 |
| **420** | *Ilex serrata* | leaves, twigs | Aquifoliaceae | Korea | SNUG | 198 |
| **421** | *Crataegus scabrida* | leaves | Rosaceae | Korea | SNUG | 204 |
| **422** | *Crataegus scabrida* | twigs | Rosaceae | Korea | SNUG | 185 |
| **423** | *Sonchus brachyotus* | flower | Compositae | Korea | SNUG | 195 |
| **424** | *Patrinia scabiosaefolia* | flower | Umbelliferae | Korea | SNUG | 185 |
| **425** | *Chrysanthemum indicum* | leaves | Compositae | Korea | SNUG | 184 |
| **426** | *Disporum viridescens* | fruits | Colchicaceae | Korea | SNUG | 200 |
| **427** | *Disporum viridescens* | leaves | Colchicaceae | Korea | SNUG | 163 |
| **428** | *Crataegus scabrida* | fruits | Rosaceae | Korea | SNUG | 138 |
| **429** | *Agrimonia pilosa* | whole | Rosaceae | Korea | SNUG | 151 |
| **430** | *Bupleurum falcatum* | flower, whole | Umbelliferae | Korea | SNUG | 120 |
| **431** | *Sium suave* | whole | Umbelliferae | Korea | SNUG | 173 |
| **432** | *Peucedanum japoincum* | whole | Umbelliferae | Korea | SNUG | 146 |
| **433** | *Heteropappus hispidus* | whole | Compositae | Korea | SNUG | 201 |
| **434** | *Angelica koreana* | whole | Umbelliferae | Korea | SNUG | 179 |
| **435** | *Echinops setifer* | fruits | Compositae | Korea | SNUG | 161 |
| **436** | *Echinops setifer* | whole | Compositae | Korea | SNUG | 141 |
| **437** | *Epimedium koreanum* | whole | Berberidaceae | Korea | SNUG | 151 |
| **438** | *Perilla frutescens var. acuta* | whole | Lamiaceae | Korea | SNUG | 121 |
| **439** | *Thymus quinquecostatus* | whole | Labiatae | Korea | SNUG | 148 |
| **440** | *Peucedanum terebinthaceum* | whole | Umbelliferae | Korea | SNUG | 134 |
| **441** | *Aster incisus* | whole | Compositae | Korea | SNUG | 127 |
| **442** | *Syneilesis palmata* | leaves | Compositae | Korea | SNUG | 124 |
| **443** | *Veronica kiusiana var. maxima* | flower | Scrophulariaceae | Korea | SNUG | 130 |
| **444** | *Cynanchum atratum* | whole | Asclepiadaceae | Korea | SNUG | 172 |
| **445** | *Caragana sinica* | whole | Fabaceae | Korea | SNUG | 178 |
| **446** | *Leonurus japonicus* | whole | Lamiaceae | Korea | SNUG | 187 |
| **447** | *Sedum aizoon* | whole | Crassulaceae | Korea | SNUG | 212 |
| **448** | *Sedum kamtschaticum* | whole | Crassulaceae | Korea | SNUG | 207 |
| **449** | *Scilla scilloides* | bulb | Asparagaceae | Korea | SNUG | 195 |
| **450** | *Scilla scilloides* | whole | Asparagaceae | Korea | SNUG | 189 |
| **451** | *Agrimonia pilosa* | root | Rosaceae | Korea | SNUG | 205 |
| **452** | *Agrimonia pilosa* | leaves | Rosaceae | Korea | SNUG | 232 |
| **453** | *Agrimonia pilosa* | stem | Rosaceae | Korea | SNUG | 214 |
| **454** | *Camelia japonica* | root | Theaceae | Korea | SNUG | 181 |
| **455** | *Lonicera insularis* | aerial | Caprifoliaceae | Korea | SNUG | 95 |
| **456** | *Lonicera insularis* | root | Caprifoliaceae | Korea | SNUG | 80 |
| **457** | *Lonicera japonica* | whole | Caprifoliaceae | Korea | SNUG | 101 |
| **458** | *Lonicera japonica* | root | Caprifoliaceae | Korea | SNUG | 98 |
| **459** | *Lonicera maackii* | aerial | Caprifoliaceae | Korea | SNUG | 94 |
| **460** | *Lonicera maackii* | root | Caprifoliaceae | Korea | SNUG | 98 |
| **461** | *Lonicera praeflorens* | aerial | Caprifoliaceae | Korea | SNUG | 75 |
| **462** | *Lonicera praeflorens* | root | Caprifoliaceae | Korea | SNUG | 89 |
| **463** | *Lonicera sachalinensis* | aerial | Caprifoliaceae | Korea | SNUG | 127 |
| **464** | *Lonicera sachalinensis* | root | Caprifoliaceae | Korea | SNUG | 98 |
| **465** | *Lonicera vesicaria* | aerial | Caprifoliaceae | Korea | SNUG | 171 |
| **466** | *Lonicera vesicaria* | root | Caprifoliaceae | Korea | SNUG | 156 |

^a^KRIBB: Korea Plant Extract Bank, Korea Research Institute of Bioscience & Biotechnology ^b^SNUG: the Medicinal Plant Garden in Seoul National University

**Supplementary Figures:**

**
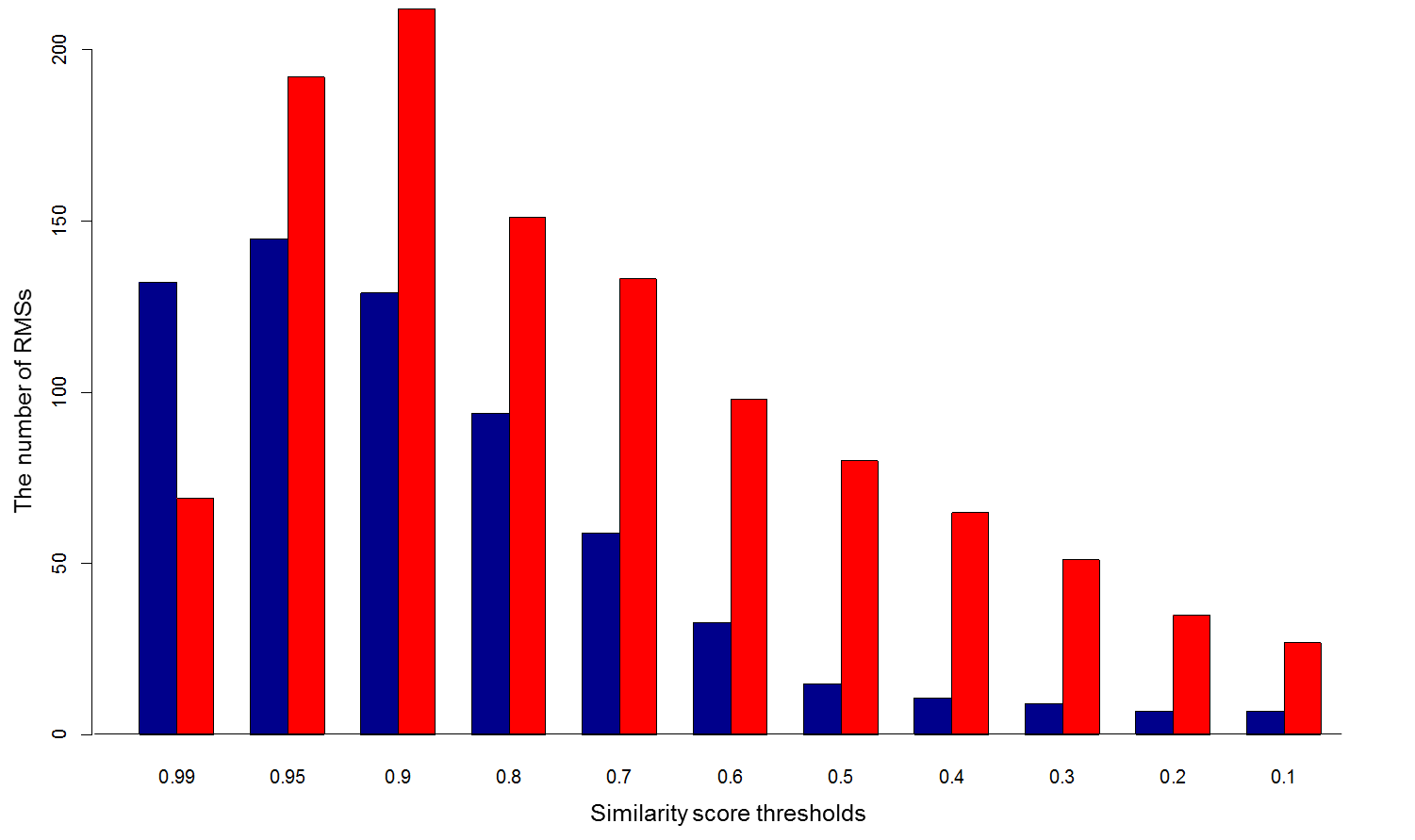
**

**Figure S1**. The number of the representative MS scans in the range of 0.1 and 099 of the similarity scores thresholds. The blue and red bars indicate the number of RMSs from *A. pilosa* roots and the aerial parts, respectively.

**
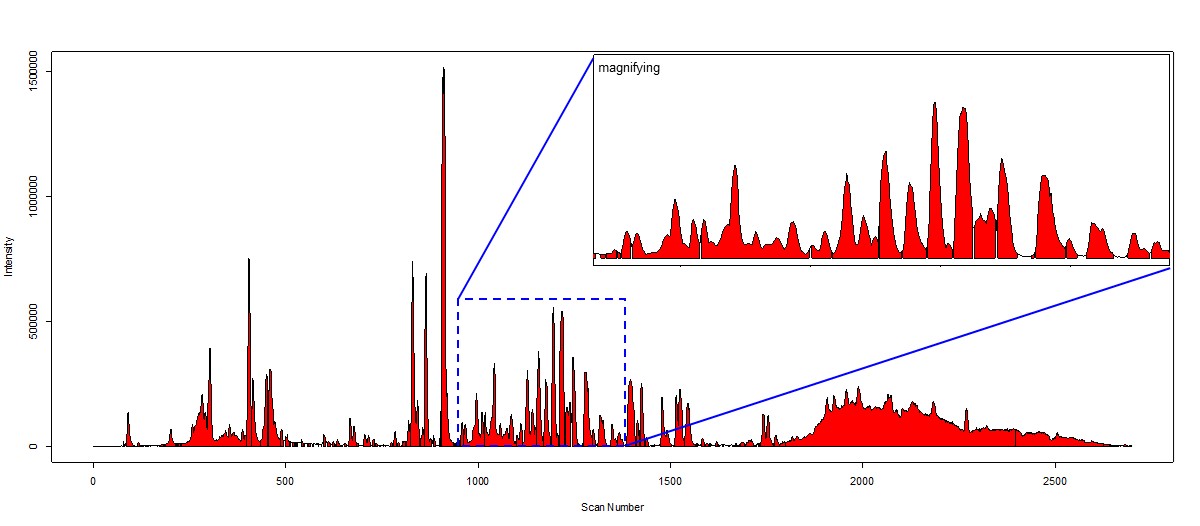
**

(a)


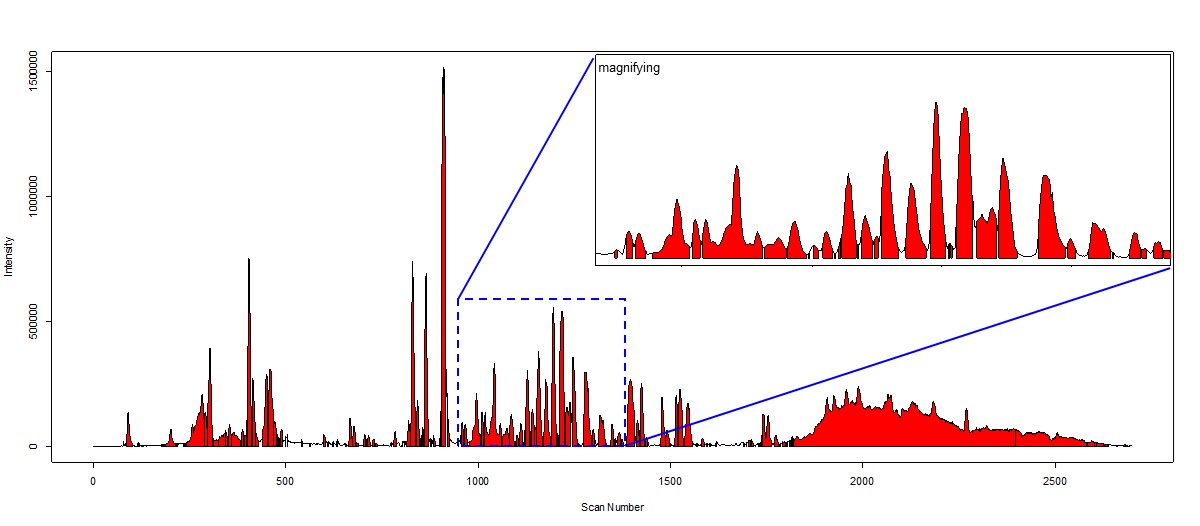


(b)

**
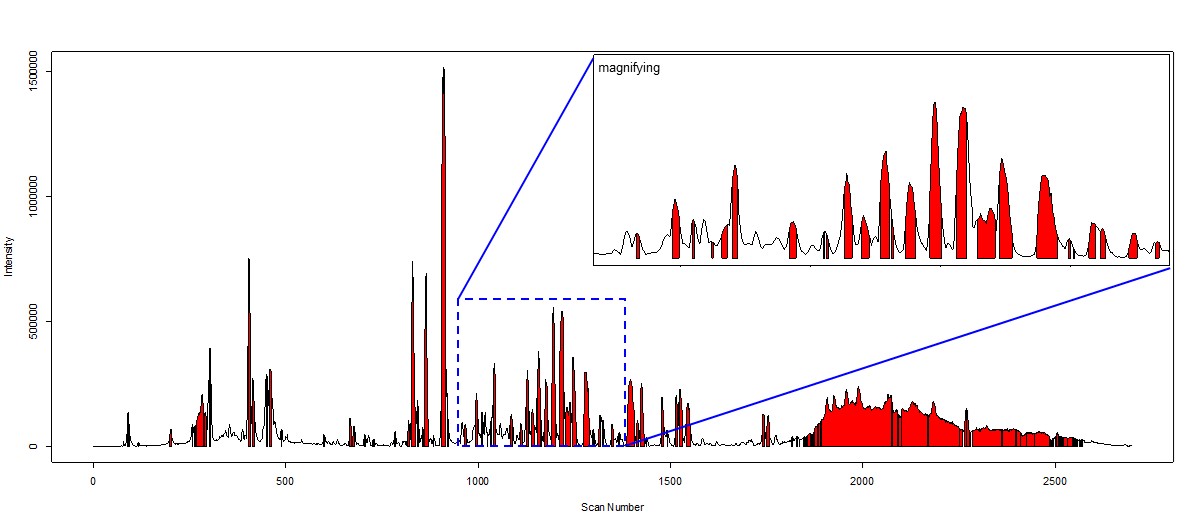
**

(c)

**
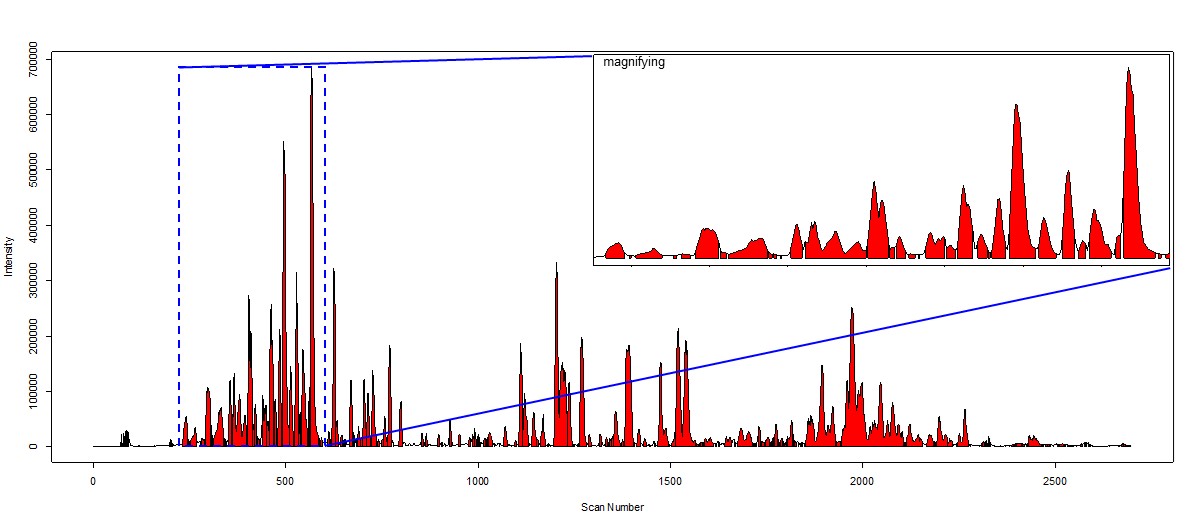
**

(d)

**
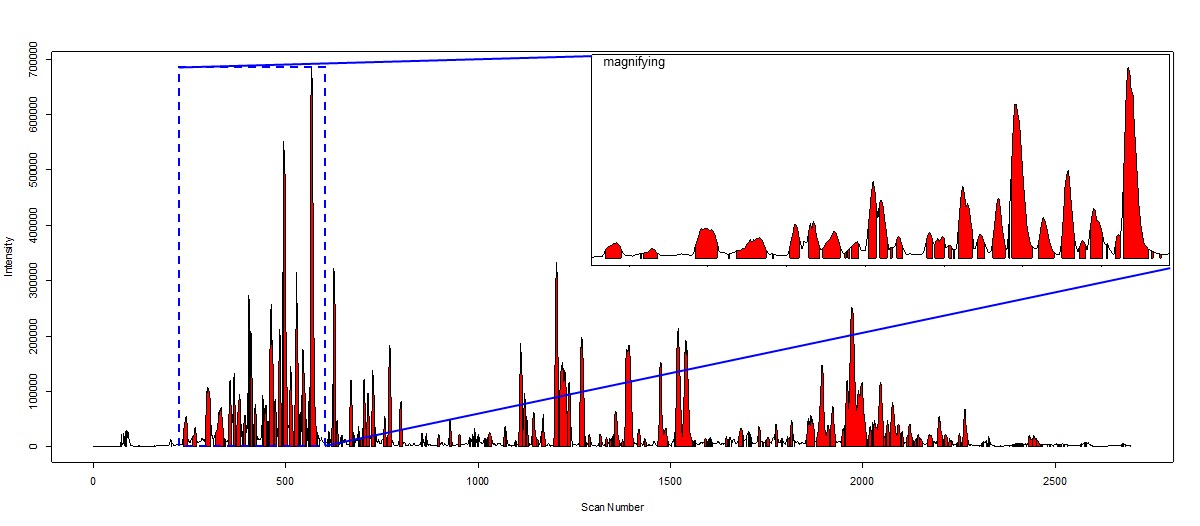
**

(e)


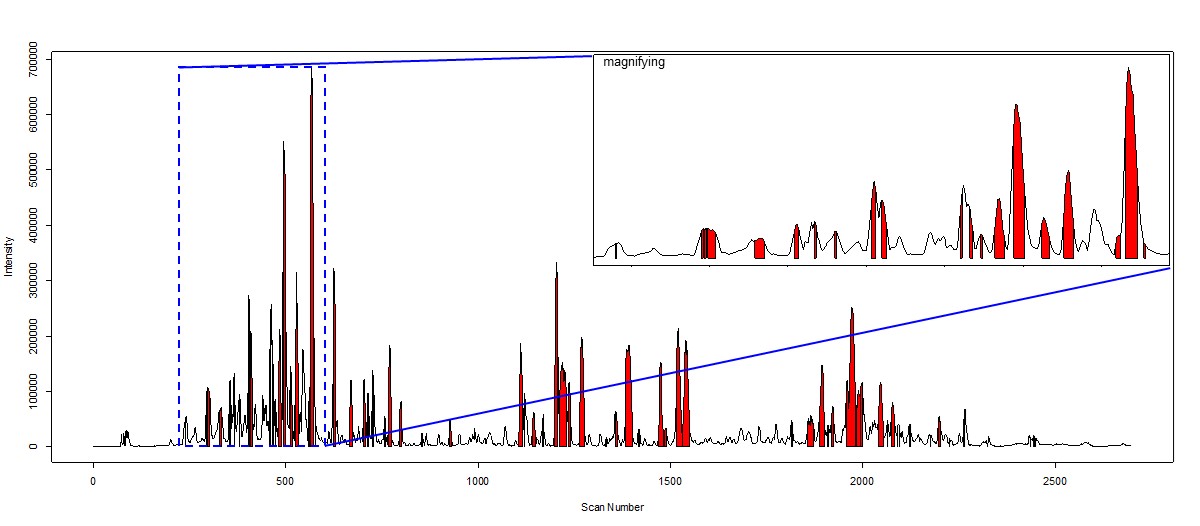


(f)

**Figure S2**. The quality of the representative MS spectra, RMSs, of *A.* *pilosa* roots (a-c) and the aerial parts (d-f) by the similarity scores thresholds. The numbers of the RMSs were 129 (a), 145 (b) and 106 (c) for *A. pilosa* roots, and 212 (d), 192 (e) and 69 (f) for the aerial parts of *A. pilosa* at the similarity score thresholds of 0.90 (a, d), 0.95 (b, e) and 0.99 (c, f), respectively.

**
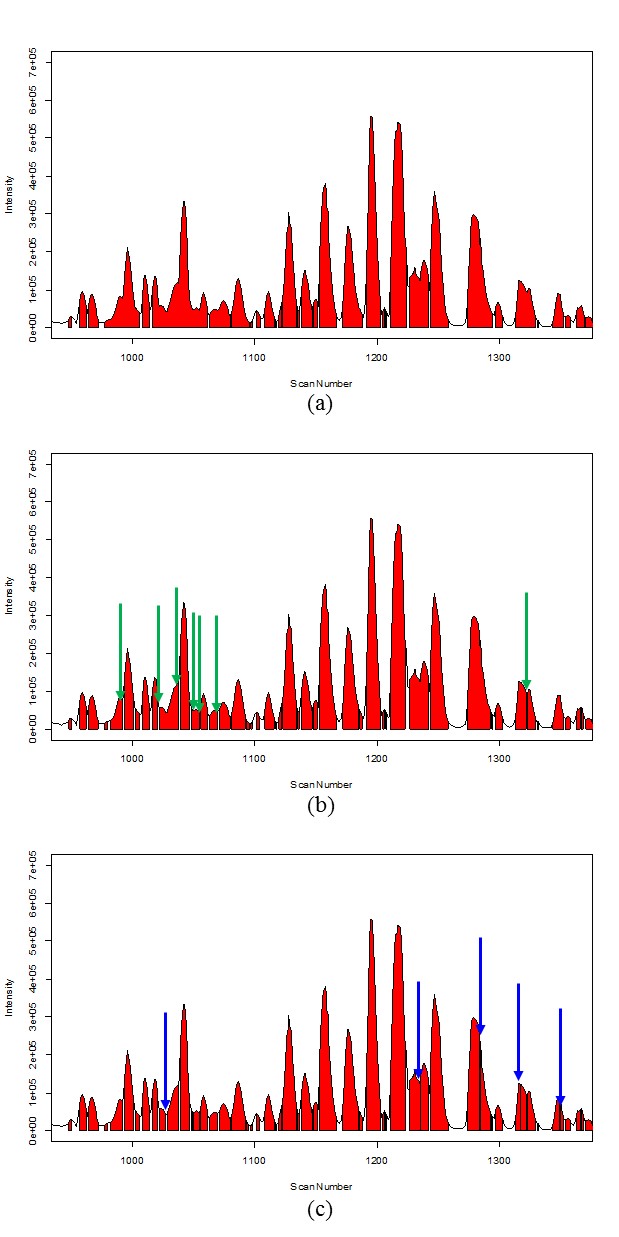
**

**Figure S3**. The improvement of the quality of the representative MS spectra (RMSs) by two deconvolution filters. The resolution of RMSs which were obtained only by the evaluation of the similarities between the consecutive MS spectra (a) was improved by two deconvolution filters, the base peak ions filter (BPI filter) (b) and the chromatographic peak shape filter (CPS filter) (c). Green arrows indicate the RMSs additionally separated from RMSs profile only generated by the similarity scoring after the application of BPI filter and blue ones the RMSs by blue ones by CPS filter, respectively.

(continue)

**Figure S4**. The RMSs of compounds **1**, **2**, **6-11** and **16-21** isolated from *A. pilosa* roots

**Figure S5**. The symmetric matrix consisting of the similarity score profiles between 189 RMSs obtained in *A. pilosa* roots for the hierarchical clustering analysis. A x_k,j_ denotes the dot-product similarity score between k_th_ RMS (S_k_) and j_th_ RMS (S_j_) in 189 RMSs of *A. pilosa* roots

**Figure S6**. The dendrogram of 189 RMSs of *A. pilosa* roots was yielded by Pearson coefficient based distance and Ward.D linkage. Arrows indicate 14 RMSs corresponding to single secondary metabolites (**1**, **2**, **6-11** and **16-21**) isolated from *A. pilosa* roots reported in the previous study^13^. Their structures are presented in Fig S7.

**Figure S7**. The structures of compounds **1**-**43** isolated from *A. pilosa* roots and the aerial parts. The procedures of the isolation and the structures determination are described in Supplementary Note 2 and 3, respectively.


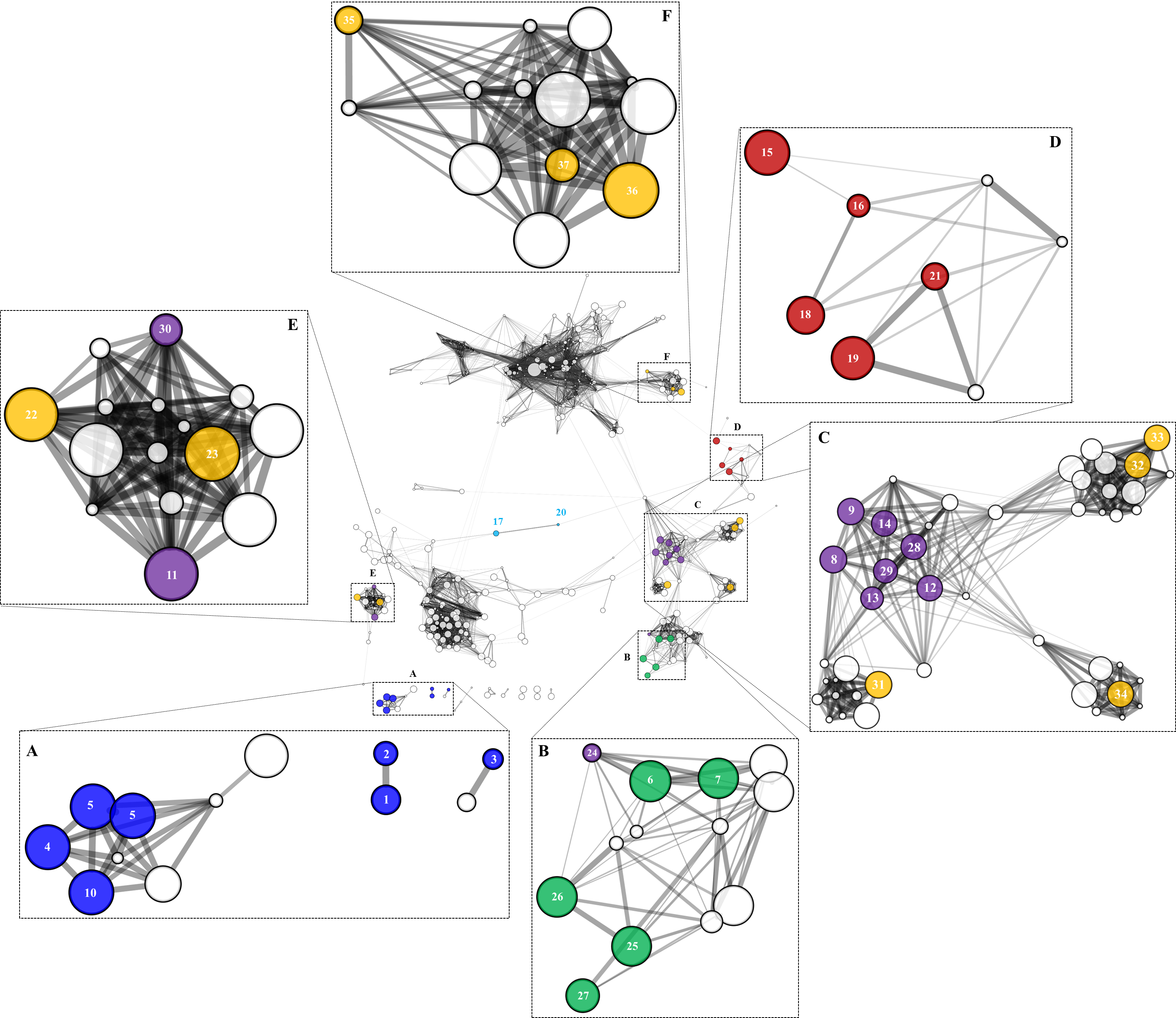


**Figure S8**. The molecular network of 246 RMSs (127 for roots and 119 for aerial parts) generated from *A. pilosa* roots and aerial parts. The similarity score threshold between RMSs is 0.5. The size of a node is the sum of ions intensities in each RMS, and the thickness of an edge proportionally represents the similarity scores of two nodes. The nodes are marked according to the types of the secondary metabolites isolated from *A. pilosa* as follows; blue: agrimolide-type (**1**-**5** and **10**), light green: pilosanidin-type (**6**, **7** and **25**-**27**), violet: pilosanol-type (**8**, **9**, **11**-**14**, **24** and **28**-**30**), red: chromone-type (**15**, **16**, **18**, **19** and **21**), sky blue: flavonoid glycoside-type (**17** and **20**) and yellow: triterpene-type (**22**, **23**, **31**-**34** and **35**-**37**). Full names of **1**-**37** are listed in Table 1.

**Figure S9**. The RMSs of **6**, **12** and **15**.

**
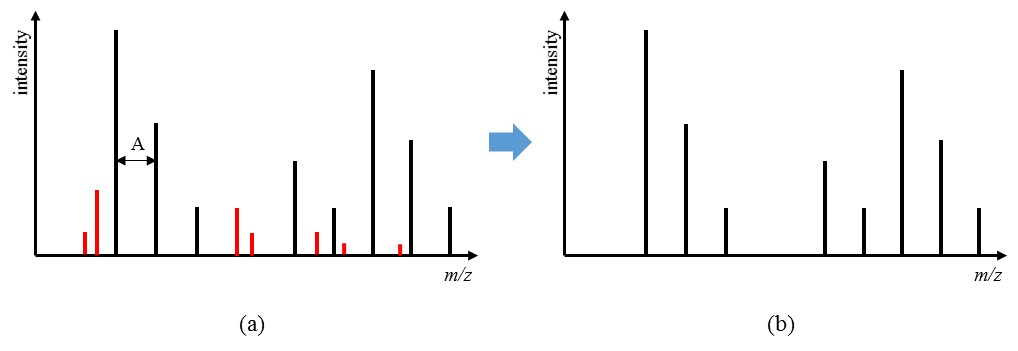
**

**Figure S10**. The overview of noise-filtering process. (a) the raw MS spectrum and (b) noise filtered MS spectrum. For extracting of noise filtered MS scans, the noisy ion peaks (red lines) with *m/z* and intensity thresholds or more are removed and the isotope patterns with the space of *m/z* 1.0033 ± 0.005 (A) were extracted.

**Figure S11**. The overview of the idealized de-isotoping procedures. (a) For an isotopic pattern with only decreasing intensities, first *m/z* with the strongest intensity and the sum of intensities were regarded as *m/z* and its intensity corresponding to the monoisotopic peak. (b) For a peak profile showing a jagged pattern due to overlapped peaks derived by more than two ions, the experimental isotope patterns were deconvoluted in the accordance with the theoretical isotope profiles calculated using 813,997 elemental formula with C, H, O, N, P and S deposited in Pubchem database (April, 2017).

**References**

1. Plumb, R. S. et al. UPLC/MS(E); a new approach for generating molecular fragment information for biomarker structure elucidation. *Rapid Commun. Mass Spectrom.* **20**, 1989-1994 (2006).

2. Bocker, S., Letzel, M. C., Liptak, Z. & Pervukhin, A. SIRIUS: decomposing isotope patterns for metabolite identification. *Bioinformatics* **25**, 218-224 (2009).

3. Kind, T. & Fiehn, O. Advances in structure elucidation of small molecules using mass spectrometry. *Bioanal. Rev.* **2**, 23-60 (2010).

4. Kuhl, C., Tautenhahn, R., Bottcher, C., Larson, T. R. & Neumann, S. CAMERA: an integrated strategy for compound spectra extraction and annotation of liquid chromatography/mass spectrometry data sets. *Anal. Chem.* **84**, 283-289 (2012).

5. Yergey, J. A. A general approach to calculating isotopic distributions for mass spectrometry. *Int. J. Mass Spectrom. Ion Phys.* **52**, 337-349 (1983).

6. Wehofsky, M. & Hoffmann, R. Automated deconvolution and deisotoping of electrospray mass spectra. *J. Mass Spectrom.* **37**, 223-229 (2002).

7. Kim, S. et al. PubChem substance and compound databases. *Nucleic Acids Res.* **44**, D1202-1213 (2016).

8. Song, H., Hsu, F. F., Ladenson, J. & Turk, J. Algorithm for processing raw mass spectrometric data to identify and quantitate complex lipid molecular species in mixtures by data-dependent scanning and fragment ion database searching. *J. Am. Soc. Mass Spectrom.* **18**, 1848-1858 (2007).

9. Frank, A. M. et al. Clustering millions of tandem mass spectra. *J. Proteome Res.* **7**, 113-122 (2008).

10. Kasai, S., Watanabe, S., Kawabata, J., Tahara, S. & Mizutani, J. Antimicrobial catechin derivatives of *Agrimonia pilosa*. *Phytochemistry* **31**, 787-789 (1992).

11. Taira, J., Ohmine, W., Ogi, T., Nanbu, H. & Ueda, K. Suppression of nitric oxide production on LPS/IFN-gamma-stimulated RAW264.7 macrophages by a novel catechin, pilosanol N, from *Agrimonia pilosa* Ledeb. *Bioorg. Med. Chem. Lett.* **22**, 1766-1769 (2012).

12. Hattori, Y. et al. A refined method for determining the absolute configuration of the 3-hydroxy-3-methylglutaryl group. *Tetrahedron-Asymmetr* **18**, 1183-1186 (2007).

13. Kim, H. W. et al. Acylphloroglucinolated catechin and phenylethyl isocoumarin derivatives from *Agrimonia pilosa*. *J. Nat. Prod.* **79**, 2376-2383 (2016).
